# Seasonal patterns in microbial carbon and iron transporter expression in the Southern Ocean

**DOI:** 10.1186/s40168-023-01600-3

**Published:** 2023-08-19

**Authors:** Pavla Debeljak, Barbara Bayer, Ying Sun, Gerhard J. Herndl, Ingrid Obernosterer

**Affiliations:** 1 Laboratoire d’Océanographie Microbienne (LOMIC), CNRS, Sorbonne Université, Banyuls/Mer, F-66650 France; 2 Department of Functional and Evolutionary Ecology, University of Vienna, Djerassiplatz 1, Vienna, 1030 Austria; 3 SupBiotech, Villejuif, France; 4 Department of Microbiology and Ecosystem Science, Centre for Microbiology and Environmental Systems Science, University of Vienna, Djerassiplatz 1, Vienna, 1030 Austria; 5 BGI-Qingdao, BGI-Shenzhen, Qingdao, 266555 China; 6 Department of Marine Microbiology and Biogeochemistry, NIOZ (Royal Netherlands Institute for Sea Research), Den Burg, 1790 AB The Netherlands; 7 Vienna Metabolomics Center, University of Vienna, Djerassiplatz 1, Vienna, 1030 Austria

**Keywords:** Prokaryotic membrane transporters, Organic substrate utilization, Iron uptake, Metatranscriptomics, Metaproteomics, Metagenome-assembled genomes, Southern Ocean, Ocean microbiome

## Abstract

**Background:**

Heterotrophic microbes in the Southern Ocean are challenged by the double constraint of low concentrations of organic carbon (C) and iron (Fe). These essential elements are tightly coupled in cellular processes; however, the prokaryotic requirements of C and Fe under varying environmental settings remain poorly studied. Here, we used a combination of metatranscriptomics and metaproteomics to identify prokaryotic membrane transporters for organic substrates and Fe in naturally iron-fertilized and high-nutrient, low-chlorophyll waters of the Southern Ocean during spring and late summer.

**Results:**

Pronounced differences in membrane transporter profiles between seasons were observed at both sites, both at the transcript and protein level. When specific compound classes were considered, the two approaches revealed different patterns. At the transcript level, seasonal patterns were only observed for subsets of genes belonging to each transporter category. At the protein level, membrane transporters of organic compounds were relatively more abundant in spring as compared to summer, while the opposite pattern was observed for Fe transporters. These observations suggest an enhanced requirement for organic C in early spring and for Fe in late summer. Mapping transcripts and proteins to 50 metagenomic-assembled genomes revealed distinct taxon-specific seasonal differences pointing to potentially opportunistic clades, such as Pseudomonadales and *Nitrincolaceae,* and groups with a more restricted repertoire of expressed transporters, such as Alphaproteobacteria and *Flavobacteriaceae*.

**Conclusion:**

The combined investigations of C and Fe membrane transporters suggest seasonal changes in the microbial requirements of these elements under different productivity regimes. The taxon-specific acquisition strategies of different forms of C and Fe illustrate how diverse microbes could shape transcript and protein expression profiles at the community level at different seasons. Our results on the C- and Fe-related metabolic capabilities of microbial taxa provide new insights into their potential role in the cycling of C and Fe under varying nutrient regimes in the Southern Ocean.

Video Abstract

**Supplementary Information:**

The online version contains supplementary material available at 10.1186/s40168-023-01600-3.

## Background

Heterotrophic prokaryotes consume roughly half of primary production and thereby influence the flux of carbon through the marine food web. The transformation of phytoplankton-derived organic matter by prokaryotes shapes the amount and quality of dissolved organic matter (DOM) that can accumulate in surface waters on a seasonal time scale and eventually be exported to depth via overturning circulation [1–3].

The concentration and composition of a variety of individual compounds that make up the pool of DOM determine its overall bioavailability and thus the quantity of carbon (C) that can be transformed over different time scales [4]. This process is regulated by inorganic nutrients essential for microbial metabolism. Nitrogen and phosphorus are the primary growth-limiting factors for heterotrophic prokaryotes in different regions of the oligotrophic ocean [1], and these nutrients might lead to seasonal accumulation of DOM in surface waters [2, 5]. In the Southern Ocean, where these macronutrients persist at high concentrations throughout the seasons, the trace element iron (Fe) is a limiting or co-limiting factor for prokaryotic growth [5–7]. These previous observations from experimental studies provide, however, restricted information on the temporal and spatial variability of the requirements of Fe by Southern Ocean prokaryotes.

Marine DOM is composed of diverse substrates of varying bioavailability. The complexity of organic compounds contained in DOM has become more accessible through advances in analytical methods [6, 7]. Concurrently, the chemical characterization and quantification of numerous siderophores have provided novel insights into Fe biogeochemistry [8]. However, organic substrate and Fe-uptake strategies of diverse prokaryotic taxa are still poorly understood. Expression profiles of microbial transporter genes can be used as an indicator to describe patterns of organic matter uptake and to link these to taxonomy [9–11]. Metaproteomic studies have revealed that a wide range of organic molecules participate in the microbial DOM flux [12–14]. Metatranscriptomics provided insights into the microbial uptake of a suite of highly labile organic substrates, including nitrogen-containing compounds such as taurine [15] and one-carbon compounds such as methanol [16], fatty acids [15], and sulfonates [17]. In a similar manner, the use of different forms of inorganic and organically bound Fe by diverse prokaryotes was illustrated [18–20]. However, combined investigations of C and Fe transporters under changing resource supply remain scarce [21, 22].

The objective of the present study was to identify seasonal differences in compound-specific transporters for C and Fe of microbial communities at contrasting sites in the Southern Ocean. Our study was carried out in the Kerguelen region where natural Fe fertilization leads to annually recurring phytoplankton blooms in otherwise high-nutrient-low-chlorophyll (HNLC) waters [23]. These blooms profoundly affect heterotrophic prokaryotic growth, activity, and community composition during different bloom stages [24–27]. We used a combined metatranscriptomic and metaproteomic approach to study C- and Fe-uptake patterns at two sites with contrasting seasonal productivity regimes in early spring and late summer, and we further mapped gene expression data to metagenomic-assembled genomes (MAGs) to link phylogeny with function.

## Methods

### Sample collection

Surface seawater samples (10 m) were collected during the Southern Ocean and Climate (SOCLIM) cruise in early spring (ES) (Oct. 6th to Nov. 1st, 2016) and during the Marine Ecosystem Biodiversity and Dynamics of Carbon around Kerguelen (MOBYDICK) cruise in late summer (LS) (Feb. 18th to Mar. 29th, 2018). Two stations were chosen for the present study: one station was located in high-nutrient low-chlorophyll (HNLC) waters (KERFIX; 50°40′ S–68°25′ E), and one station was located in naturally iron-fertilized waters above the central plateau of Kerguelen (A3; 50°38′ S–72°02′ E) (Supplementary Fig. 1A). Both stations were visited two to three times during both cruises, except for station KERFIX which was sampled only once during the SOCLIM campaign (Table 1). Seawater was sampled with Niskin bottles mounted on a CTD frame and transferred into 10–20 L polycarbonate carboys using acid-washed tubing and a 60-µm nylon screen. Extractions were performed with the same kits and methods for both cruises, and sequencing depth was kept the same for all replicates (supplementary material; Supplementary Table 1).

**Table 1:** Brief description of the study sites. Early spring sampling was carried out during the SOCLIM cruise in 2016, and late summer sampling was carried out during the MOBYDICK cruise in 2018. All values are from surface waters (20 m). DOC, Chl *a* and prokaryotic abundance (PA) data in early spring are from Liu et al. (2019) [26] and in late summer from Hernandez et al. (2021) [27]

**Station**	**Date**	**Temp (°C)**	**DOC (µM)**	**Chl *a* (µg L**^**−1**^**)**	**PA (× 10**^**8**^** L**^**−1**^**)**
**Early spring**					
A3_1	18 Oct	2.19	52	1.44	3.66
A3_2	24 Oct	2.06	51	1.64	4.88
KERFIX	18 Oct	2.38	51	0.32	2.89
**Late summer**					
A3_1	26 Feb	5.01	53	0.27	11.8
A3_2	6 Mar	5.24	55	0.32	8.37
A3_3	17 Mar	5.26	53	0.57	6.65
KERFIX_1	3 Mar	5.62	50	0.19	6.96
KERFIX_2	20 Mar	5.63	49	0.14	4.46

### Metagenomics

Sampling and metagenomics analyses, including the construction of metagenome-assembled genomes (MAGs), are described elsewhere [21] (supplementary material; Supplementary Fig. 2; Supplementary Tables 2, 3 and 4). Briefly, 6-L seawater from each station was pre-filtered through 0.8-µm membrane filters (Isopore, Millipore) using a 47-mm filtration system and further collected on a 0.2-µm Sterivex cartridge (Millipore). DNA was extracted from each Sterivex filter unit using the AllPrep DNA/RNA kit (Qiagen, Hilden, Germany). DNA purification was performed following the manufacturer’s guidelines, and DNA quality was checked on an Agilent 2100 Bioanalyzer/Agilent Nano DNA chip (Agilent, Santa Clara, CA, USA). Shotgun library preparation was performed by Fasteris SA using the Illumina Nano Library Preparation Kit with 550-bp size selection. Each metagenome was sequenced on one full lane of HiSeq 4000 with 150-bp paired-end reads yielding between 285 and 339 million reads per metagenome. Decontaminated, trimmed and normalized metagenomic sequences were co-assembled using MEGAHIT v1.0.4 [28] with the default parameters and the –presets “meta-large” option resulting in 949,228 contigs of at least 1000 bp (Supplementary Tables 2 and 3). Prodigal was used for annotating open reading frames (ORFs) in the metagenomic mode (-meta) [29]. The MetaWRAP pipeline was implemented to recover individual genomes from the assembled contigs with three binning tools, including CONCOCT [30], MaxBin v2.0 [31] and MetaBAT v2.0 [32], and yielded 133 MAGs. The completeness and redundancy of each MAG were estimated by CheckM [33]. Taxonomic classification of the 133 MAGs was determined by the classify_wf workflow of the GTDB-Tk toolkit [34] based on the Genome Taxonomy Database (GTDB v0.3.0) and further confirmed by phylogeny inference based on single-copy orthologous gene families (Supplementary Table 4) [21]. We have additionally performed antiSMASH v5.1.2 as recent application on ocean microbiome data and transporter proteins [35, 36] in order to identify biosynthetic clusters of our MAGs and have added the information in Suppl. Tables 11 and 12.

### Metatranscriptomics

For RNA extractions, 10-L seawater were pre-filtered through 0.8-µm membrane filters (Isopore, Millipore), and cells were collected on 0.2-µm membranes (SuporPlus, Millipore) using a 142-mm filtration system (Geotech Equipment Inc.) and a peristaltic pump. RNA was extracted using the NucleoSpin® RNA Midi kit (Macherey-Nagel, Düren, Germany; supplementary material) according to the manufacturer’s protocol. The extracted RNA was quantified and quality checked using an Agilent 2100 Bioanalyzer/Agilent RNA 6000 Nano Kit (Agilent, Santa Clara, CA, USA). Prior to sequencing, ribosomal RNA was treated enzymatically with the RiboZero rRNA-stranded RNA protocol to ensure sequencing of primarily messenger RNA followed by cDNA library construction using Illumina TruSeq-stranded mRNA Library Prep kit (Fasteris SA). The rRNA percentages varied between 0.6 and 2.6% of total reads per library after sortmeRNA and the RiboZero rRNA depletion kit that were used by Fasteris. All metatranscriptomes (*n* = 12) were sequenced on one lane of HiSeq 4000 with 150-bp paired-end reads yielding between 26 and 36 million reads per metatranscriptome (Supplementary Table 5). Quality-filtered metatranscriptomic coding sequences were mapped against the metagenomic co-assembly, and the resulting count tables obtained for spring and summer samples were compared (supplementary material; Supplementary Table 6). Differential expression analysis was performed with DESeq2 (v1.24.0) [37] to identify transcripts with significant changes in relative abundance (adjusted *p*-value < 0.05). Additionally, ALDEx2 [38] was used to verify with a second differential abundance method as suggested by Nearing et al. [39], and results are provided in Suppl. Tables 13 and 14.

### Metaproteomics

For protein extractions, 20-L seawater were pre-filtered through 0.8-µm filter membranes (Isopore, Millipore), and cells were collected on 0.2-µm filter membranes (SuporPlus, Millipore) using a 142-mm filtration system (Geotech Equipment Inc.) and a peristaltic pump. Filtrations were performed in duplicate for both seasons and frozen immediately at −80 °C. Whole protein extractions from filters were performed using a modified protocol from Bayer et al. [40], and extracted proteins were subjected to denaturing polyacrylamide gel electrophoresis (SDS-PAGE) followed by overnight trypsin in-gel digestion (supplementary material). Peptides were extracted and desalted using 96-well plates (SPEC 96-well C18, Agilent) and then resuspended in 2% acetonitrile/0.1% formic acid to a concentration of 0.2 µg µL^−1^ prior to injection into a one-dimensional nanoflow LC–MS/MS (supplementary material, Supplementary Table 7). The normalized spectral abundance factor (NSAF) was used as a proxy for relative protein abundances and was calculated as follows [41]:

$${NSAF}_{k}={\left(\frac{PSM}{L}\right)}_{k}/\sum_{i=1}^{N}{\left(\frac{PSM}{L}\right)}_{i}$$

where the total number of spectral counts for the matching peptides from protein *k* (PSM) was divided by the protein length (*L*) and then divided by the sum of PSM/*L* for all *N* proteins.

### Protein database construction and annotation of transporter proteins

A total of 3,003,586 protein-coding genes were identified from the assembled metagenomic contigs by Prodigal as mentioned above. Protein sequences were clustered by CD-HIT-2D [42, 43] (-c 0.9 -n 5 -d 0 -S 2) to eliminate redundancy. The resulting nonredundant proteins were pooled with the Global Ocean Sampling (GOS) amino acid sequence database in order to include more protein sequences from the marine environment, stemming from longer reads and thus more robust for the purpose of the analysis [44]. Another round of cd-hit clustering (-c 1 -n 5 -d 0) was performed to remove identical amino acid sequences resulting in 58,403,522 sequences (Supplementary Fig. 2). In order to identify genes encoding transporter proteins in the individual metagenomic assemblies, predicted amino acid sequences were queried against the KEGG database with GhostKOALA [42] and eggNOG5.0 [40, 41] using eggnog-mapper v2 [45] with default parameters. Sequences classified as “transporter” in the KEGG database were retrieved from metatranscriptomes and metaproteomes (provided as electronic supplemental material). Transporter families and compound specificities were verified by manually checking the KO number with assigned classification from the Transporter Classification Database [46–49]. For definite assignment of a protein sequence with a MAG, the sequences corresponding to transporters were aligned against the high-quality curated metagenomic bins, and those with 95% identity and 90% coverage were kept for MAG-specific further analysis. To focus specifically on carbon and Fe utilization, we used existing hidden Markov models for carbohydrate-active enzymes (CAZymes) ([50]; http://www.cazy.org/) and Fe-specific transport (Supplementary Tables 8 and 9) [51]. To identify potential siderophore producers, we further searched for BGC on the MAG using antiSMASH (v5.1.2) [52], and the respective results are presented in the Supplementary Tables 11 and 12).

## Results and discussion

### Biogeochemical characteristics of the study sites

Natural fertilization supplies waters above the Kerguelen plateau continuously with low quantities of Fe, thereby stimulating seasonal primary production and associated food web processes within otherwise HNLC waters [23]. For the present study, samples were collected in early spring at the onset of the annually recurring phytoplankton bloom and in late summer under post-bloom conditions (Supplementary Fig. 1B & C). Concentrations of chlorophyll *a* (Chl *a*) were ≈ 5-fold higher at the on-plateau station A3 (1.54 μg L^−1^) as compared to station KERFIX (0.32 μg L^−1^) in HNLC waters in early spring (Table 1). These differences were far less pronounced during late summer (≈ 0.39 μg L^−1^ at A3 and ≈ 0.17 μg L^−1^ at KERFIX). An opposite seasonal pattern was observed for prokaryotic abundances at both sites with about 2-fold higher cell abundances in late summer (8.94 × 10^8^ cells L^−1^ at A3 and 5.71 × 10^8^ cells L^−1^ at KERFIX) than in early spring (4.27 × 10^8^ cells L^−1^ at A3 and 2.89 × 10^8^ cells L^−1^ at KERFIX) [26, 27]. Above the Kerguelen plateau, we observed slightly higher dissolved organic carbon (DOC) concentrations in late summer (54 ± 1 µmol L^−1^) as compared to spring (52 ± 1 µmol L^−1^). Despite the continuous input of Fe, dissolved Fe concentrations were not substantially different between sites, an observation that can be explained by the rapid biological utilization of Fe in surface waters [23]. On the seasonal scale, dissolved Fe concentrations were higher in early spring (0.16 nM at A3 and 0.13 nM at KERFIX) [53] than in summer (0.09 nM and 0.07 nM at A3 and KERFIX, respectively) [54].

### Transporters for organic carbon and iron

A total of 144 and 156 transporter transcripts and proteins were identified by metatranscriptomics (MT) and metaproteomics (MP), respectively, of which 98 transporters were shared between the two datasets. The proportion of transporters made up between 3.5 and 12.3% of the total normalized transcript counts in the metatranscriptomes, whereas their proportion in the metaproteomes ranged from 42.8 to 96.8% (Supplementary Fig. 3). Membrane transporters belonging to the ATP-binding cassette transporter family (ABC) had the highest contribution (MT range 2.8 to 10.8%; MP range 21.2 to 88.9%), followed by outer-membrane receptors (OMR) (MT range 0.22 to 0.77%; MP range 3.6 to 19.6%) (Supplementary Fig. 3). A similar distribution pattern of these transporter types was observed previously in surface waters of East Antarctica [55] and other ocean regions and depth layers [12, 56, 57].

**Fig. 1:**
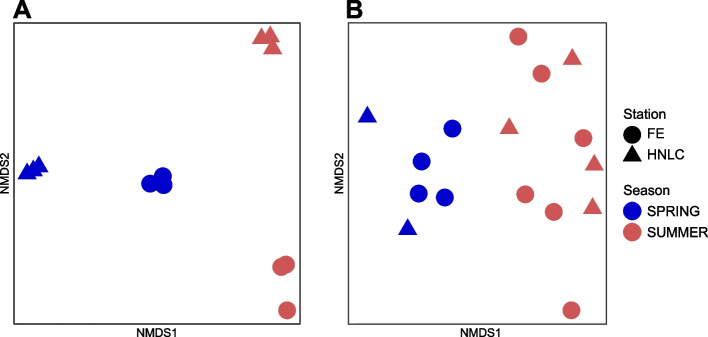
Nonmetric multidimensional scaling (NMDS) of transporter proteins in **A** metatranscriptomes and **B** metaproteomes based on Hellinger-transformed Bray–Curtis dissimilarity values. Biological replicates are shown for each station and revisit (detailed in Table S1). Samples from the two seasons were significantly different (metatranscriptomes: 1D stress = 0.00001, ANOSIM, *R* = 0.88, *p* < 0.001; metaproteomes: 2D stress = 0.1; ANOSIM, *R* = 0.84, *p* = 0.001)

**Fig. 2:**
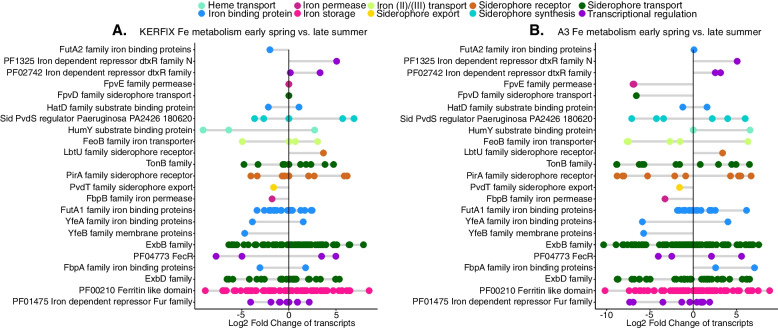
Community-level seasonal differences in transcript abundances of transporters at station KERFIX (**A**) and A3 (**B**). Each dot represents transcripts for which log2fold changes could be obtained in differential expression analysis. Negative values represent transcripts that are less abundant in late summer than in early spring; positive values represent transcripts that are more abundant in late summer than in early spring

Membrane transporter profiles were significantly different between seasons in both the MT and MP datasets (ANOSIM, *p* < 0.001) (Fig. 1). We further explored these patterns for each of the MT and MP dataset. Transcripts encoding for protein families related to Fe transport showed significant differences in their relative abundances between both seasons (Fig. 2).

However, most protein families (with the exception of PF01325 and PF02742 related to transcriptional regulation of Fe metabolism) contained transcripts that either increased or decreased in relative abundance. As a consequence, no seasonal trend in Fe-related transport proteins was observed in the MT data (Fig. 2). Similarly, the proportions of transporters for organic substrates were not different between spring and summer (Supplementary Fig. 4).

**Fig. 3:**
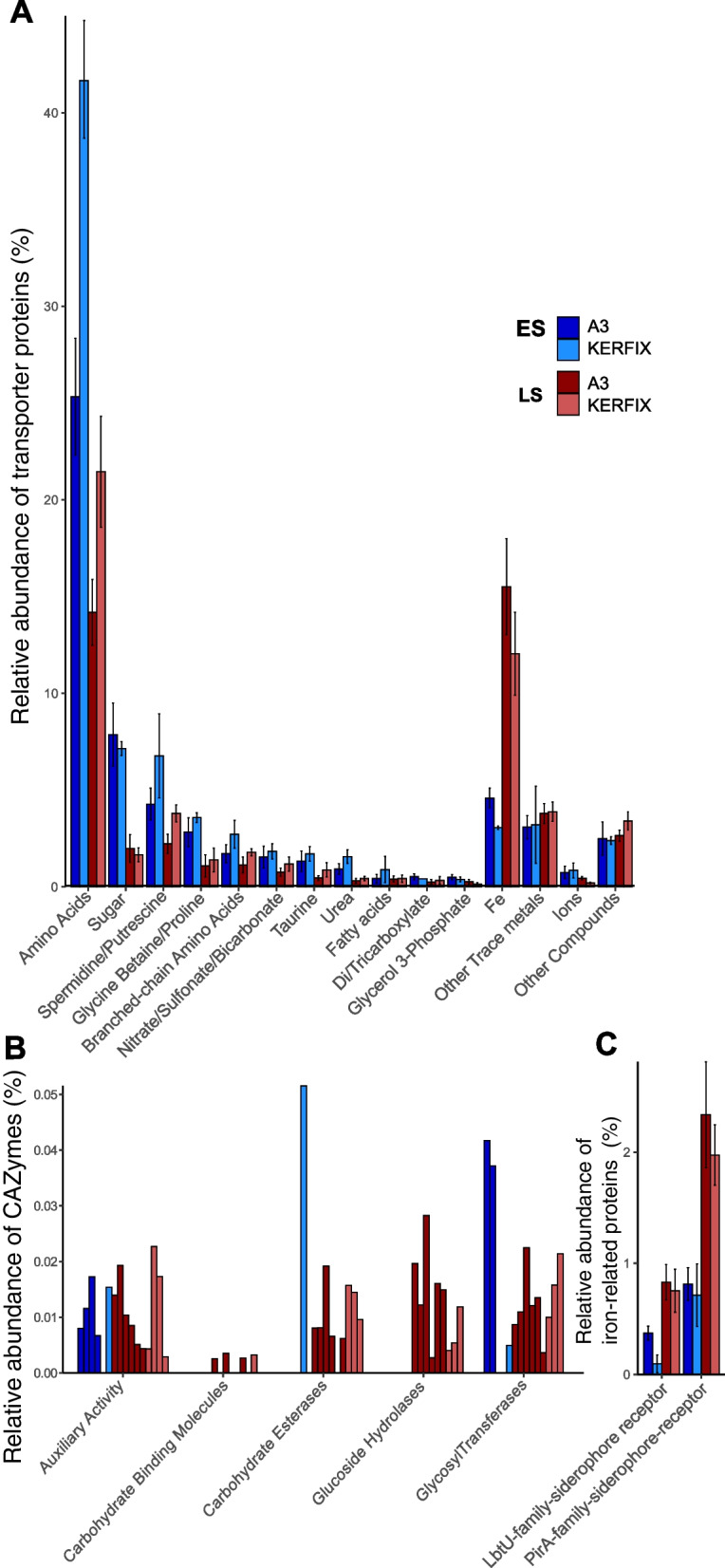
Relative abundances of transporter proteins and carbohydrate-active enzymes in metaproteomes. Bars show mean values with error bars as minimum and maximum values (except for **B** where all samples are plotted) from 2 metaproteomes. ES, early spring; LS, late summer. **A** Compound-specific transporters. **B** Carbohydrate-active enzymes (CAZymes). **C** Iron siderophore transport proteins (classified with FeGenie)

A contrasting observation was made on the protein level. Most of the Fe-transporter proteins showed higher relative abundances in summer than in spring at both sites, while an opposite pattern was observed for transporters of organic C compounds (Fig. 3).

Transporters for substrates containing both carbon and nitrogen were most abundant and were dominated by amino acid transporters, which accounted for 25–42% and 15–21% of the identified transporter proteins in early spring and late summer, respectively, at the two sites. A similar seasonal decrease was observed for transporters of spermidine/putrescine (6.8% in spring to 2.2% in summer), glycine betaine/proline (3.6 to 1.1%) and branched-chain amino acids (2.7 to 1.1%).

Transporters for taurine, urea, fatty acids and nitrate/sulfonate/bicarbonate were low in relative abundance (< 2%) during both seasons at both sites (Fig. 3A). Transporters for sugars accounted for 7.9% in spring and 1.6% in summer. Auxiliary activities, which are occurring in conjunction with CAZymes such as carbohydrate esterases, glucoside hydrolases and glycosyl transferases, were detected at both sites and seasons accounting for < 0.05% of total identified proteins (Fig. 3B). However, CAZymes were lacking pronounced differences between spring and late summer. Transporters for Fe (ABC and OMR) exhibited higher relative abundances in late summer (15.5% of total identified transporters) than in early spring (4.6%) at both sites (Fig. 3A). Similarly, the two specific siderophore receptors LbTU and PirA exhibited a 2- to 4-fold increase in their relative proportions from spring to summer (Fig. 3C). To infer patterns between organic C and Fe uptake, we calculated the ratio between the proportions of C-transporters to that of Fe transporters (sum of all organic substrate- vs. Fe-specific transporters). This ratio was substantially higher in spring (22 and 10 in HNLC and Fe-fertilized waters, respectively) than in late summer (3 and 1 in HNLC and Fe-fertilized waters, respectively).

The marked seasonal pattern in the proportion of transporters of organic substrates and Fe in the metaproteomes could reflect a switch in prokaryotic requirements of these elements between early spring and late summer. This temporal shift was observed at both sites despite the differences in the seasonal productivity regimes between fertilized and HNLC waters (Supplementary Fig. 1BC) [24]. In early spring, the onset of phytoplankton activity provides access to biologically labile DOC, a major constraint for heterotrophic prokaryotic growth during the preceding unproductive winter period [58]. The investment of the respective transporter proteins essential for the scavenging of a variety of compounds [59] illustrates the rapid response of prokaryotes to changes in the bioavailable pool of organic matter.

The seasonally high concentrations of dissolved Fe present in early spring in surface waters and the Fe stored within prokaryotic cells could both meet the prokaryotic requirements at this time of the year. In late summer, the higher prokaryotic abundances and metabolic activities [27] indicate a relief of C-limitation. In contrast, concentrations of Fe are lower in summer than in spring, and remineralization is the main form of Fe input [60–62]. The increased proportion of siderophore receptors (Fig. 3C) and transcriptional regulation (Fig. 2) suggests that the utilization of organically bound Fe plays an important role. Siderophore synthesis is energetically costly [63–65]. However, when nutrients such as carbon and nitrogen that constitute building blocks of the siderophore are in relative excess as is probably the case in late summer, organically bound Fe could become the dominant source of Fe [66]. The growth of prokaryotic taxa with the metabolic capabilities for the biosynthesis and uptake of siderophore-bound Fe could be favoured during this time period.

However, when these elements are in relative excess as they are probably in late summer, organically bound Fe could become the dominant source of iron, favouring prokaryotic taxa with the metabolic capabilities for the biosynthesis and uptake of siderophore-bound Fe [18–20].

### Reconciling metatranscriptomic and metaproteomic results

Combined mRNA and proteome investigations are expected to provide profound insight into cell physiology as they target gene transcription and translation into proteins, respectively. However, studies that concurrently apply both methods for environmental prokaryotic communities are scarce. In the present study, we observed seasonally distinct transporter profiles in metatranscriptomes and -proteomes (Fig. 1), but on the level of specific compound classes, the two approaches lead to different results (Figs. 2 and 3, Supplementary Fig. 4). Inherent properties and methodological issues should be considered for the interpretation of these results. Prokaryotic mRNA half-life times are short and highly variable (ranging from 1 to 46 min [67]), and mRNA is about an order of magnitude less abundant than DNA as well as four orders of magnitude less abundant than proteins [68]. There is a temporal decoupling that needs to be considered as proteins persist in a bacterial cell longer than the mRNA that encodes them [69–72]. In addition, while half-life times are on average higher in protein data, there might be continuous new production for both entities as well, thus the ’standing stock’ might not be influenced by half-times only. Post-transcriptional as well as post-translational regulation are also processes that can explain why mRNA levels do not correlate with protein abundance [73, 74], however, we consider that these likely play a minor role. Methodological aspects further include a generally higher throughput and resolution of RNA sequencing compared to mass spectrometry-based shotgun proteomics.

These technical aspects are reflected in our dataset. Membrane transporters contributed the majority of proteins in metaproteomes but showed substantially lower relative contributions in metatranscriptomes (Supplementary Fig. 3). An overall comparison of the metatranscriptome and metaproteome datasets showed a qualitative agreement regarding expression differences of functionally annotated genes (Supplementary Figs. 5 and 6). However, relative abundances of transcripts differed greatly from those of their corresponding proteins, as has been found in earlier studies [59, 69]. Taken together, the different patterns observed in the present study might reflect post-translational regulation involving protein modifications and proteolysis potentially leading to accumulation of specific proteins. Our data suggest that a large number of genes are differentially expressed at both seasons, but only a subset of these gene transcripts was recovered in the metaproteomes and can only in part be explained by the higher detection limit of the method.

### Differential gene expression by active members of the community

We then investigated the extent to which seasonal patterns in community-level expression of transporter proteins are detectable at the level of individual taxa. To link the expression of transporters of organic substrates and Fe to prokaryotic taxa, metagenome-assembled genomes (MAGs) were constructed, and gene sequences encoding for transporter proteins recovered from metatranscriptome and metaproteome datasets were mapped to metagenomic bins (completeness >= 50%, redundancy <= 10%; see supplementary material). A total of 133 high-quality MAGs spanning 11 phyla were obtained [21]. Gene transcripts encoding transporter proteins could be mapped to most MAGs (122 out of 133), while proteomic peptides of transporter proteins could be mapped to 50 MAGs (Supplementary Fig. 7). This is likely a result of the higher RNA-sequencing depth used in our study providing a higher sensitivity compared to mass spectrometry-based analyses of peptides as described before. We retrieved all transporter proteins present in both datasets that could be mapped to MAGs and used the respective transcripts for differential expression analyses (Supplementary material).

**Fig. 4:**
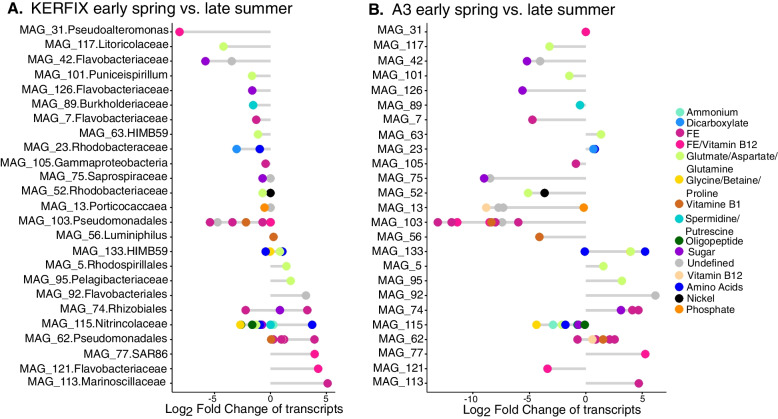
Taxon-specific seasonal differences in transcript abundances of transporters at station KERFIX (**A**) and A3 (**B**). Negative values represent transcripts that are less abundant in late summer than in early spring; positive values represent transcripts more abundant in late summer than in early spring. MAG ID shows the lowest identifiable phylogenetic level

Significant differences in transcript abundances of transporters were observed for 25 MAGs when comparing spring and summer communities, and these seasonal changes were taxon specific (Fig. 4 and Supplementary Fig. 8). Within the same MAG, either transporters for C or Fe, but not both transporter types, showed differential abundance patterns, with the exception of one MAG belonging to Rhizobiales. The highest numbers of transcripts coding for transporter proteins, which were significantly different between seasons, were associated with Pseudomonadales and *Nitrincolaceae*. Among these, Fe transporters of Pseudomonadales MAG 103 exhibited the most pronounced decrease in relative abundance from spring to summer at both stations. In contrast, the closely related Pseudomonadales MAG 62 showed the opposite pattern with higher Fe transporter abundances at both sites in late summer (Fig. 4).

**Fig. 5:**
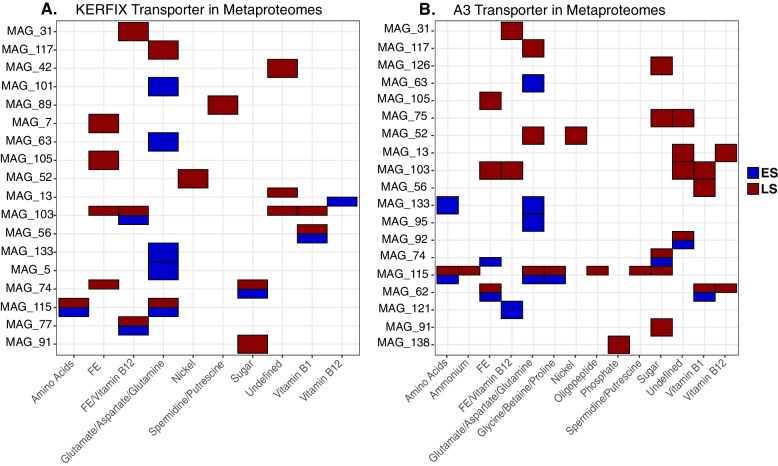
The presence of transporter proteins for different compounds in individual MAGs for KERFIX (**A**) and A3 (**B**)

The majority of transcripts of *Nitrincolaceae* MAG 115 encoding transporters for various organic C substrates including amino acids had higher relative abundances in late summer. In contrast, transcripts of amino acid transporters assigned to MAG 133 (Alphaproteobacteria HIMB59) had higher relative abundances in early spring. The role of these taxa in the expression of transporters for Fe (MAGs 103 and 62) and organic C substrates (MAG 115, MAG 133) in spring and summer was confirmed in the metaproteome dataset (Fig. 5). This view of the seasonal expression of membrane transporters illustrates the diverse ecological strategies for C and Fe acquisition among taxa and how they could shape expression profiles at the community level (Fig. 2).

Our results point to potentially opportunistic clades, such as Pseudomonadales and *Nitrincolaceae* [13, 22], and groups with a more restricted repertoire of expressed transporters, such as Alphaproteobacteria and *Flavobacteriaceae*. The differential expression of either C- and Fe- transporters could point to a temporal decoupling between cellular requirements and supply of the respective elements. In the case of C-transporters, the capability to make use of Fe stored in bacterioferritin, low cellular Fe quota or Fe-sparing metabolisms could be the underlying mechanisms [20, 75, 76]. The differential expression of Fe transporters, while that of organic substrates remain unchanged, could be due to adaptations in cellular C-metabolism [75, 77] or efficient Fe-scavenging strategies independent of organic matter supply. The pattern observed here between early spring and late summer most likely also occurs on shorter time scales.

## Conclusions

In concert, our results provide taxon-specific patterns of C and Fe transporters at two distinct stations for early spring and late summer conditions in the Southern Ocean pointing towards the complex interplay of genes, transcripts and proteins. Reconciling metatranscriptomic and metaproteomic data remains a challenging effort due to the different throughput and resolution of each approach. The interpretation using environmental data leads us to a choice of supply and demand of compounds which has yet to be clarified. Recent microfluidics studies have been used to elucidate these fine-tuned processes on a single-cell level using metagenomics [78]; however, these are currently not possible for in situ metaproteomics, due to low sample recovery [78]. The measurement of in situ uptake rates of diverse compounds by microbial cells remains a major challenge in the study of the ocean microbiome. A better understanding of these taxon-specific traits and their integration at the community level represents a major future challenge, in particular if we want to understand the responses of microbes to a changing environment.

### Supplementary Information

**Additional file 1: Supplementary Figure 1.** A Bathymetry of the Kerguelen plateau. Position along depth gradients of station KERFIX (1707m) and A3 (527m). B. Climatology of Chlorophyll *a* over 10 years at station A3 and C. station KERFIX. The green line indicates the 10-year mean; the blue line indicates the year 2018 (MOBYDICK cruise). Note different scales on y-axis.

**Additional file 2: Supplementary Figure 2.** Bioinformatic pipeline for the analysis of all three ‘omics’ levels. A. Metagenomic assembly (individual and Co-Assembly) and binning B. Construction of Ocean protein database from metagenomic assemblies C. Metatranscriptomic mapping to metagenomes after extraction of mRNA with SortMeRNA D. Metaproteomic analysis with metagenomic database and annotation in eggNOG and KEGG.

**Additional file 3: Supplementary Figure 3.** Relative proportions of transporter families. Bar-plot showing the relative proportions of different transporter families according to the transporter database (TBDB) for A. Metaproteomes from NSAF and B. Metatranscriptomes based on the total normalized transcripts. All duplicates and triplicates are shown, note difference in scale. ES -early spring, LS –late summer, Transporter types are indicated by different colors: ABC – ATP-binding cassette transporter complex, AMT/NPP – Ammonium Channel Transporters/Nitrate, Nitrate Porters, CUT – Carbohydrate Uptake Transporters, OMP – Outer Membrane Proteins, OMR – Outer Membrane Receptors, SSS – Solute Sodium Symporters, TRAP-T - Tripartite ATP-independent periplasmic transporters, TTT – Tripartite Tricarboxylate Transporters.

**Additional file 4: Supplementary Figure 4.** Compound-specific transporter transcripts. ES: Early spring; LS: Late summer. Relative proportion of compound-specific transporter transcripts of tranporters present in metatranscriptomes (see Methods section). Mean values with SD from 3 metatranscriptomes.

**Additional file 5: Supplementary Figure 5.** Heatmap of shared KEGG transporter proteins. All metatranscriptomes are shown in triplicates. Zscaling of normalized transcript counts by rows and Euclidian clustering by row and column. The presence of proteins in metaproteomes is defined by their presence in one duplicate. Transporter types are indicated by different colors: ABC – ATP-binding cassette transporter complex, AMT/NPP – Ammonium Channel Transporter/Nitrate, Nitrate Porter, CUT – Carbohydrate Uptake Transporter, OMP – Outer Membrane Protein, OMR – Outer Membrane Receptor, SSS – Solute Sodium Symporter, TRAP-T - Tripartite ATP-independent periplasmic transporters, TTT – Tripartite Tricarboxylate Transporter.

**Additional file 6: Supplementary Figure 6.** Heatmap of all identified KEGG transporter proteins. All metatranscriptomes are shown in triplicates. Z-scaling of normalized transcript counts by rows and Euclidian clustering by row and column. Presence in metaproteome datasets refers to the identified protein in at least one duplicate.

**Additional file 7: Supplementary Figure 7.** Phylogenetic tree of 50 metagenomes assembled genomes from the Co-Assembly with additional information on the expression of transporter proteins in metatranscriptomes and metaproteomes. Tree calculated from 163 single-copy genes. Additional layer represents presence of specific transporter type in MT- Metatranscriptomes and MP – Metaproteomes.

**Additional file 8: Supplementary Figure 8.** Differentially expressed transporter protein during early spring and late summer for A. Station A3 and B. Station KERFIX. Left side values represent expression in early spring and on the right side, values represent expression in late summer metatranscriptomes by transporter type. MAG Ids show the lowest identifiable phylogenetic level.

**Additional file 9: **Supplementary material.

**Additional file 10: Supplementary Table 1.** Sample and replicate description. ES – Early spring, LS – Late summer. **Supplementary Table 2.** Detailed information on metatranscriptomic libraries. **Supplementary Table 3.** Detailed information on mapping results of each metatranscriptomic library to Co-Assembly. **Supplementary Table 4.** Information on libraries used for metagenomic assembly. **Supplementary Table 5.** Outcome of Co-Assembly. **Supplementary Table 6.** Description of 133 Metagenome assembled genomes by GTDB taxonomy. **Supplementary Table 7.** Results of Mass Spectrometry Analysis for Metaproteomes. **Supplementary Table 8.** Relative normalised abundance for Metaproteome results of CAZymes HMM analysis. **Supplementary Table 9.** Relative normalised abundance for Metaproteome results of FeGenie HMM analysis. **Supplementary Table 10.** DESeq2 results. **Supplementary Table 11.** Results for Biosynthetic gene clusters (BGC) from MAG contigs. **Supplementary Table 12.** Results for Biosynthetic gene clusters from MAG contigs - siderophore related. **Supplementary Table 13.** DESeq2 results for spring vs. summer for iron-related proteins. **Supplementary Table 14.** Aldex2 results for spring vs. summer comparison.

## Data Availability

All bioinformatic procedures are detailed in Supplementary material and Supplementary Fig. 2. The data sets generated and analysed in the current study are available in the European Nucleotide Archive (ENA) repository at https://www.ebi.ac.uk/ena under the project IDs PRJEB37465 (metagenomes) and PRJEB37466 (metatranscriptomes). All acquired raw spectrum files and proteomic result files, including identified peptides, and relative protein abundances are available on MassIVE (https://massive.ucsd.edu). For MOBYDICK cruise samples proteomic result files can be found under accession number MSV000091032 (ftp://massive.ucsd.edu/MSV000091032) and under MSV000091034 (ftp://massive.ucsd.edu/MSV000091034) for SOCLIM cruise samples. All figures were produced using the ggplot2 package in R version 3.6.0 (2019-04-26) [79], and colours were enhanced in the open-source software Inkscape. Figure 3 was produced using Anvi’o visualization tool version 5.2.0 in the anvio-interactive manual mode [80].
