# Supplementary figures and images for: Seasonal patterns in microbial carbon and iron transporter expression in the Southern Ocean
Source: Microbiome. 2023 Aug 19;11:187. doi: 10.1186/s40168-023-01600-3 (PMC10439609; doi:10.1186/s40168-023-01600-3)

(A)

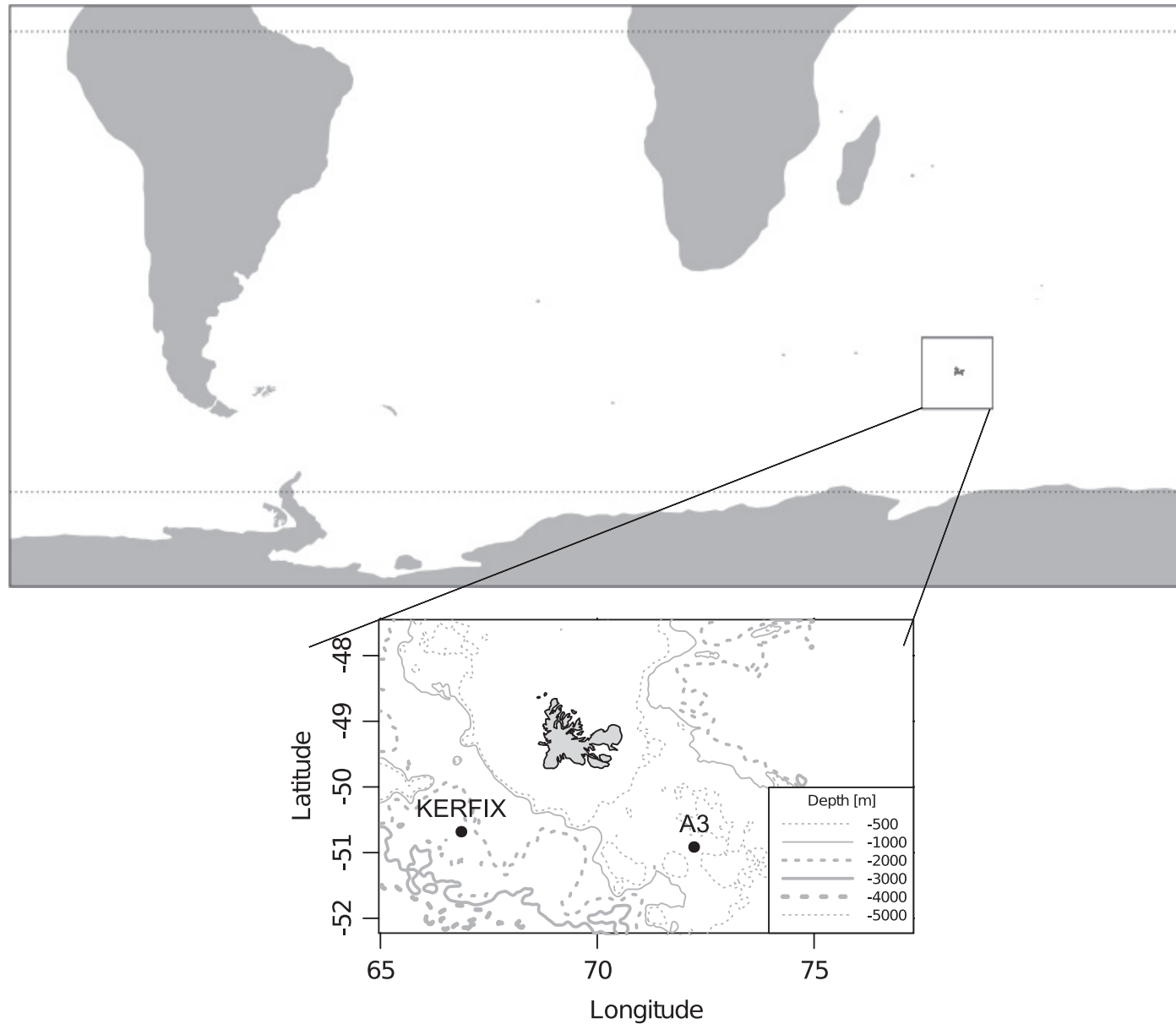

(B) Climatology Station A3

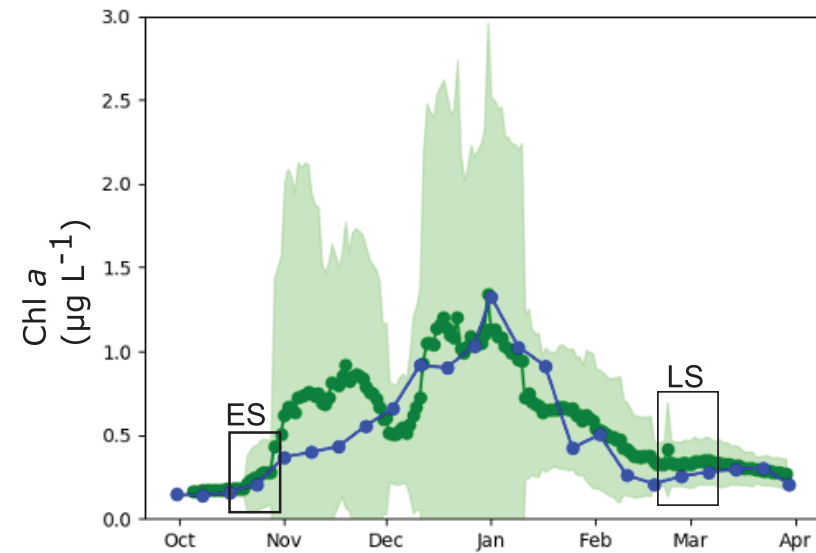(C) Climatology Station Kerfix<sub>x</sub>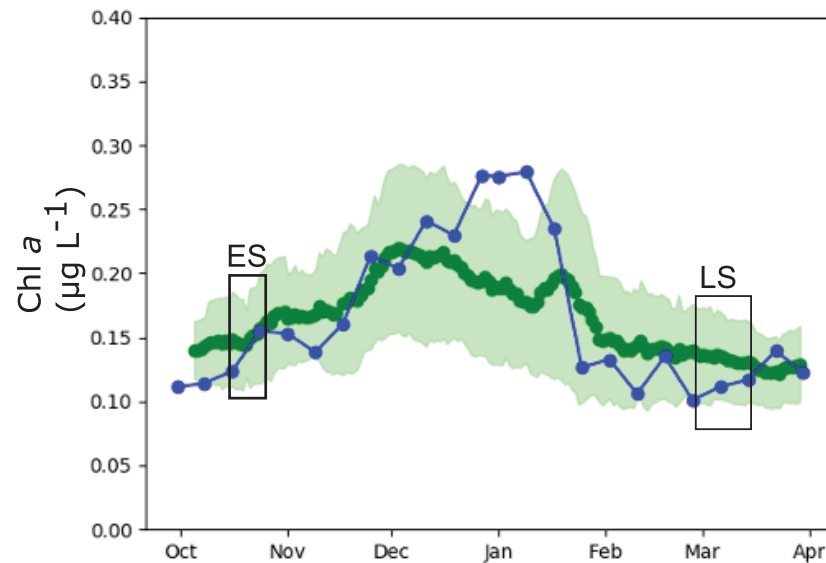

Supplement: Supplementary file 2 — Additional file 1: Supplementary Figure 1. A Bathymetry of the Kerguelen plateau. Position along depth gradients of station KERFIX (1707m) and A3 (527m). B. Climatology of Chlorophyll a over 10 years at station A3 and C. station KERFIX. The green line indicates the 10-year mean; the blue line indicates the year 2018 (MOBYDICK cruise). Note different scales on y-axis. [file 40168_2023_1600_MOESM1_ESM.pdf]

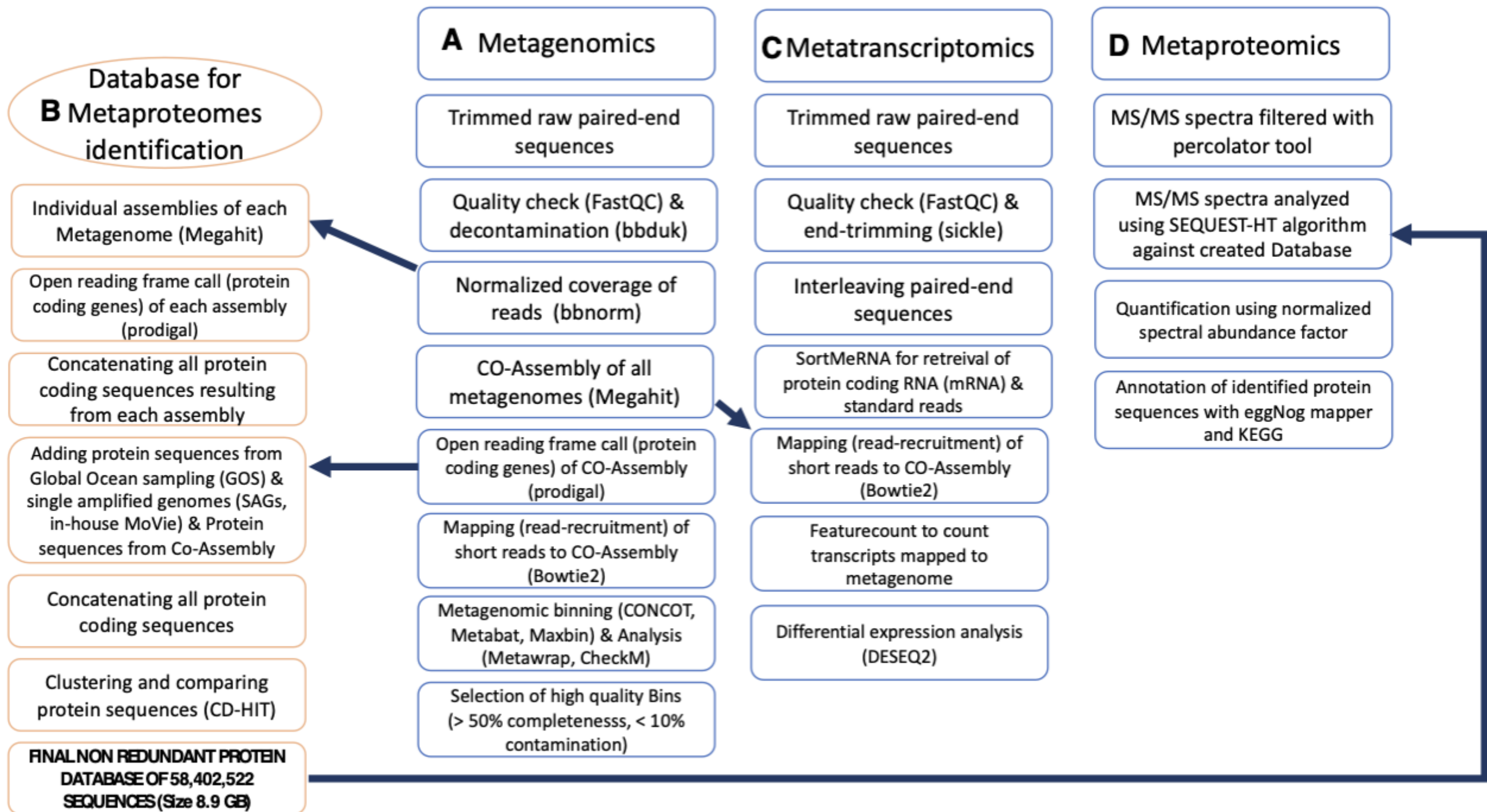

Supplement: Supplementary file 3 — Additional file 2: Supplementary Figure 2. Bioinformatic pipeline for the analysis of all three ‘omics’ levels. A. Metagenomic assembly (individual and Co-Assembly) and binning B. Construction of Ocean protein database from metagenomic assemblies C. Metatranscriptomic mapping to metagenomes after extraction of mRNA with SortMeRNA D. Metaproteomic analysis with metagenomic database and annotation in eggNOG and KEGG. [file 40168_2023_1600_MOESM2_ESM.pdf]

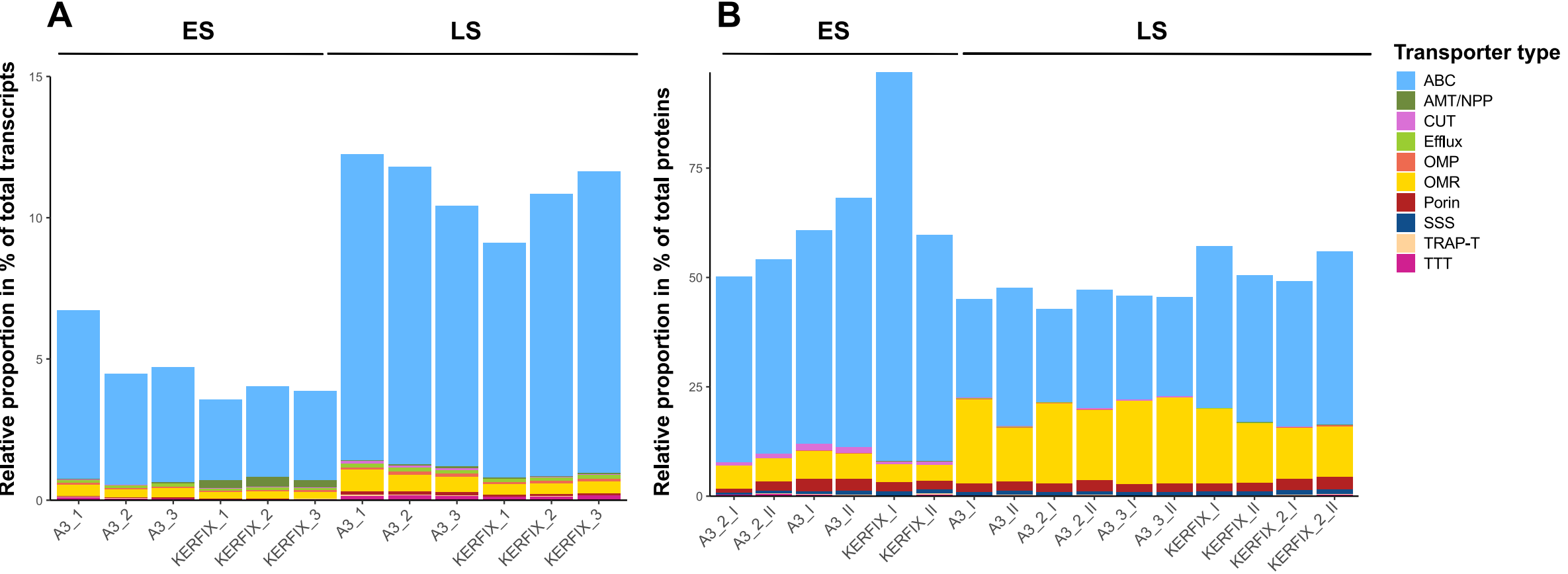

Supplement: Supplementary file 4 — Additional file 3: Supplementary Figure 3. Relative proportions of transporter families. Bar-plot showing the relative proportions of different transporter families according to the transporter database (TBDB) for A. Metaproteomes from NSAF and B. Metatranscriptomes based on the total normalized transcripts. All duplicates and triplicates are shown, note difference in scale. ES -early spring, LS –late summer, Transporter types are indicated by different colors: ABC – ATP-binding cassette transporter complex, AMT/NPP – Ammonium Channel Transporters/Nitrate, Nitrate Porters, CUT – Carbohydrate Uptake Transporters, OMP – Outer Membrane Proteins, OMR – Outer Membrane Receptors, SSS – Solute Sodium Symporters, TRAP-T - Tripartite ATP-independent periplasmic transporters, TTT – Tripartite Tricarboxylate Transporters. [file 40168_2023_1600_MOESM3_ESM.pdf]

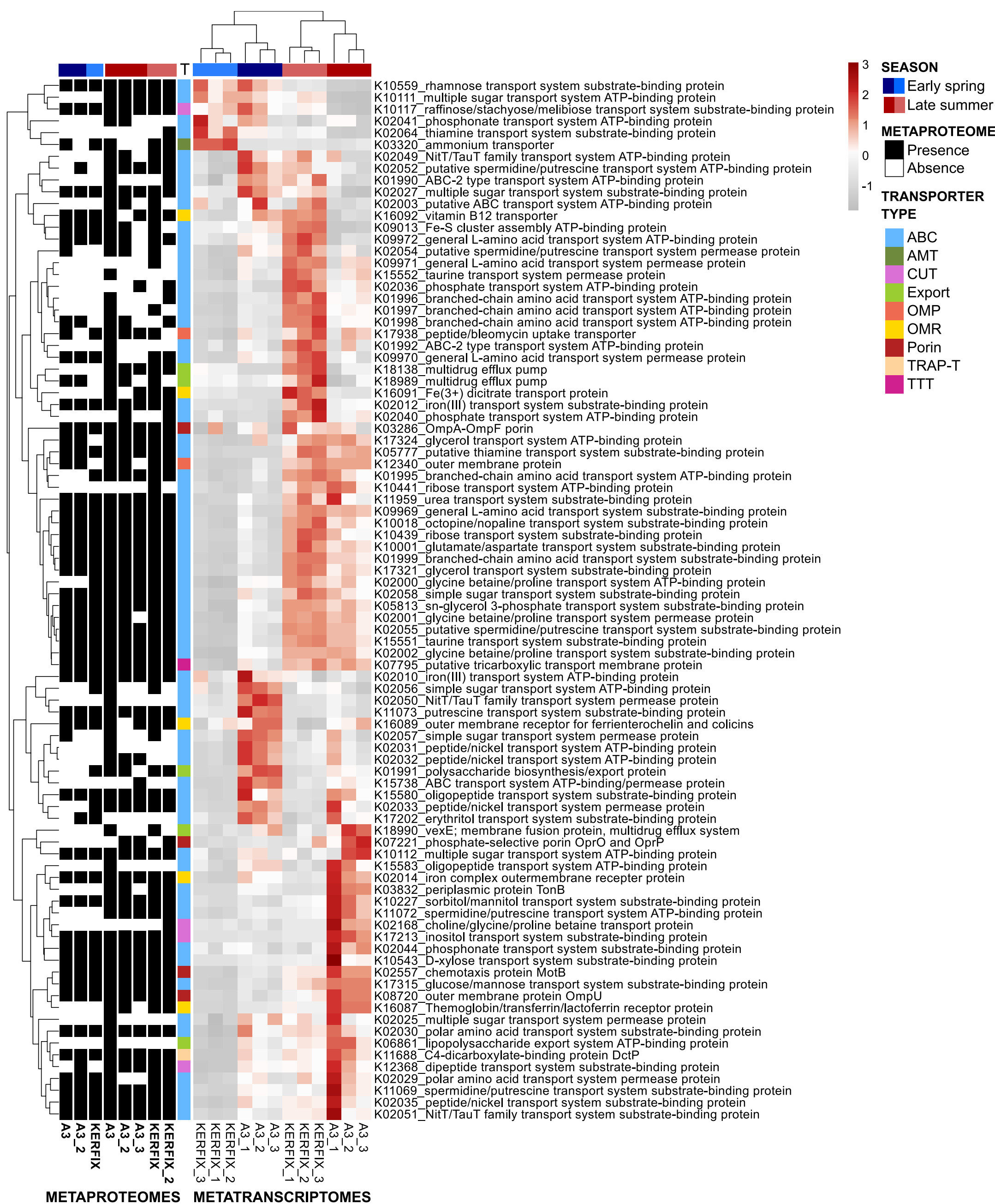

Supplement: Supplementary file 6 — Additional file 5: Supplementary Figure 5. Heatmap of shared KEGG transporter proteins. All metatranscriptomes are shown in triplicates. Zscaling of normalized transcript counts by rows and Euclidian clustering by row and column. The presence of proteins in metaproteomes is defined by their presence in one duplicate. Transporter types are indicated by different colors: ABC – ATP-binding cassette transporter complex, AMT/NPP – Ammonium Channel Transporter/Nitrate, Nitrate Porter, CUT – Carbohydrate Uptake Transporter, OMP – Outer Membrane Protein, OMR – Outer Membrane Receptor, SSS – Solute Sodium Symporter, TRAP-T - Tripartite ATP-independent periplasmic transporters, TTT – Tripartite Tricarboxylate Transporter. [file 40168_2023_1600_MOESM5_ESM.pdf]

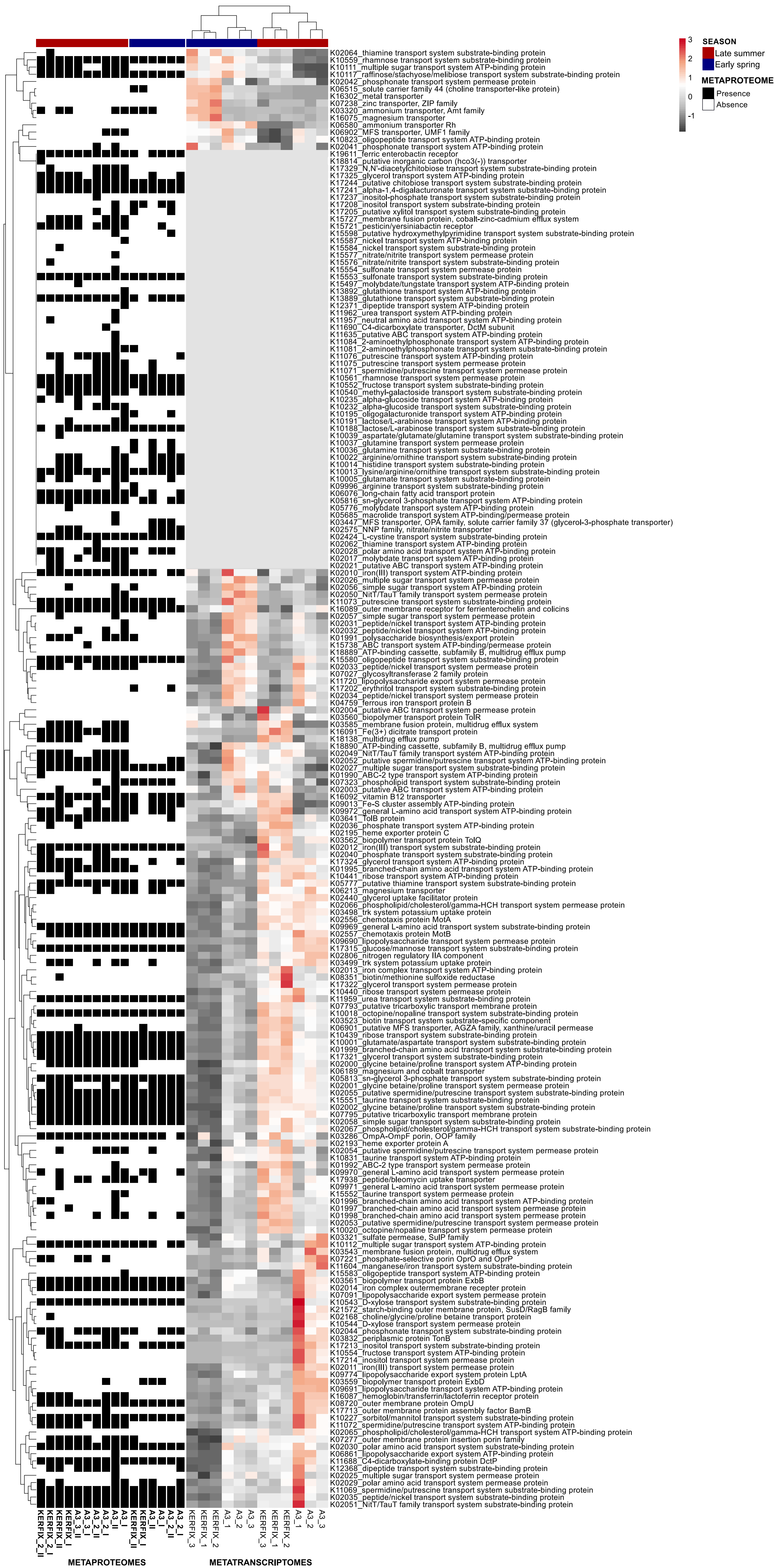

Supplement: Supplementary file 7 — Additional file 6: Supplementary Figure 6. Heatmap of all identified KEGG transporter proteins. All metatranscriptomes are shown in triplicates. Z-scaling of normalized transcript counts by rows and Euclidian clustering by row and column. Presence in metaproteome datasets refers to the identified protein in at least one duplicate. [file 40168_2023_1600_MOESM6_ESM.pdf]

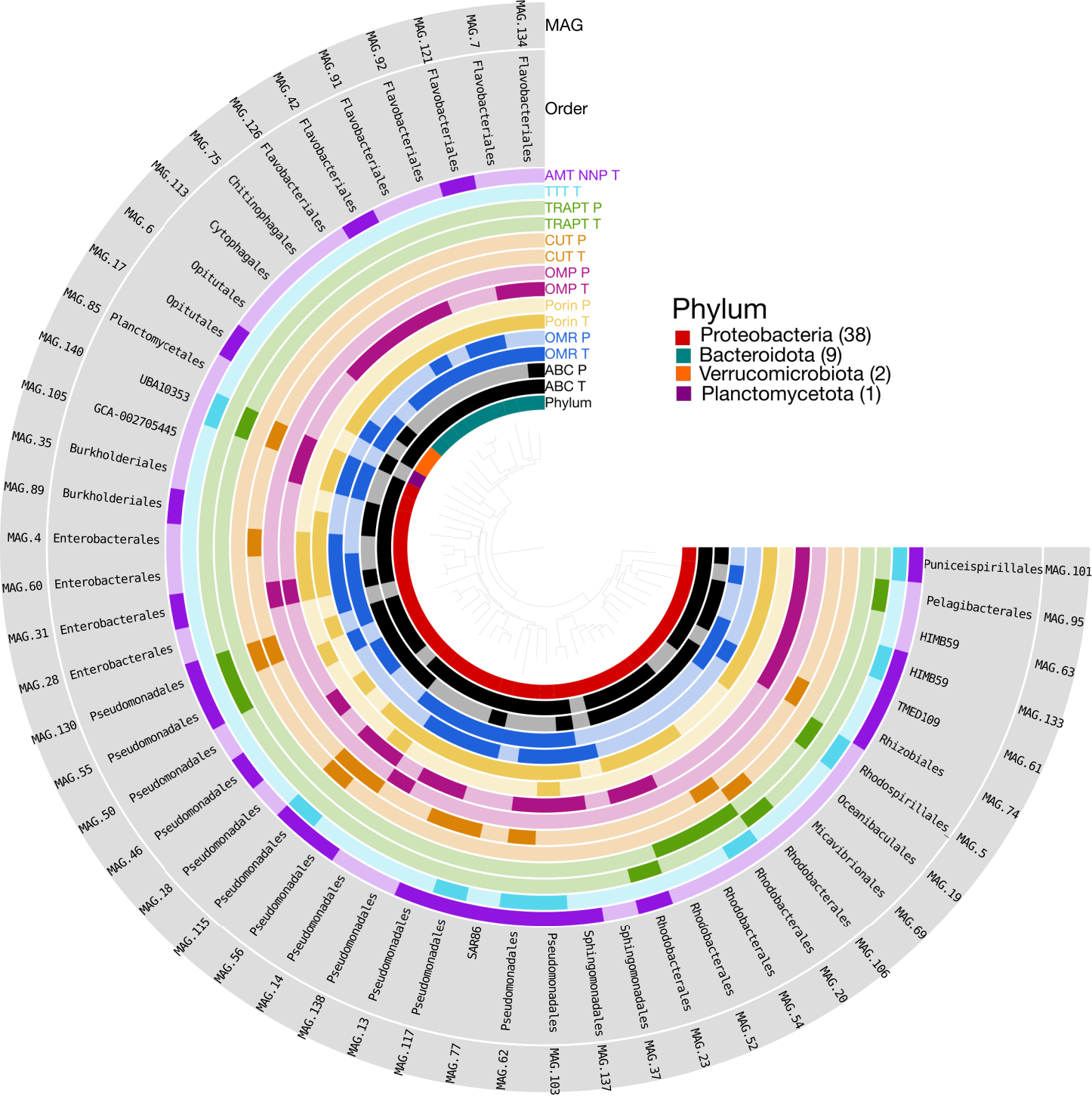

Supplement: Supplementary file 8 — Additional file 7: Supplementary Figure 7. Phylogenetic tree of 50 metagenomes assembled genomes from the Co-Assembly with additional information on the expression of transporter proteins in metatranscriptomes and metaproteomes. Tree calculated from 163 single-copy genes. Additional layer represents presence of specific transporter type in MT- Metatranscriptomes and MP – Metaproteomes. [file 40168_2023_1600_MOESM7_ESM.pdf]

**A** Station A3 Spring-Summer

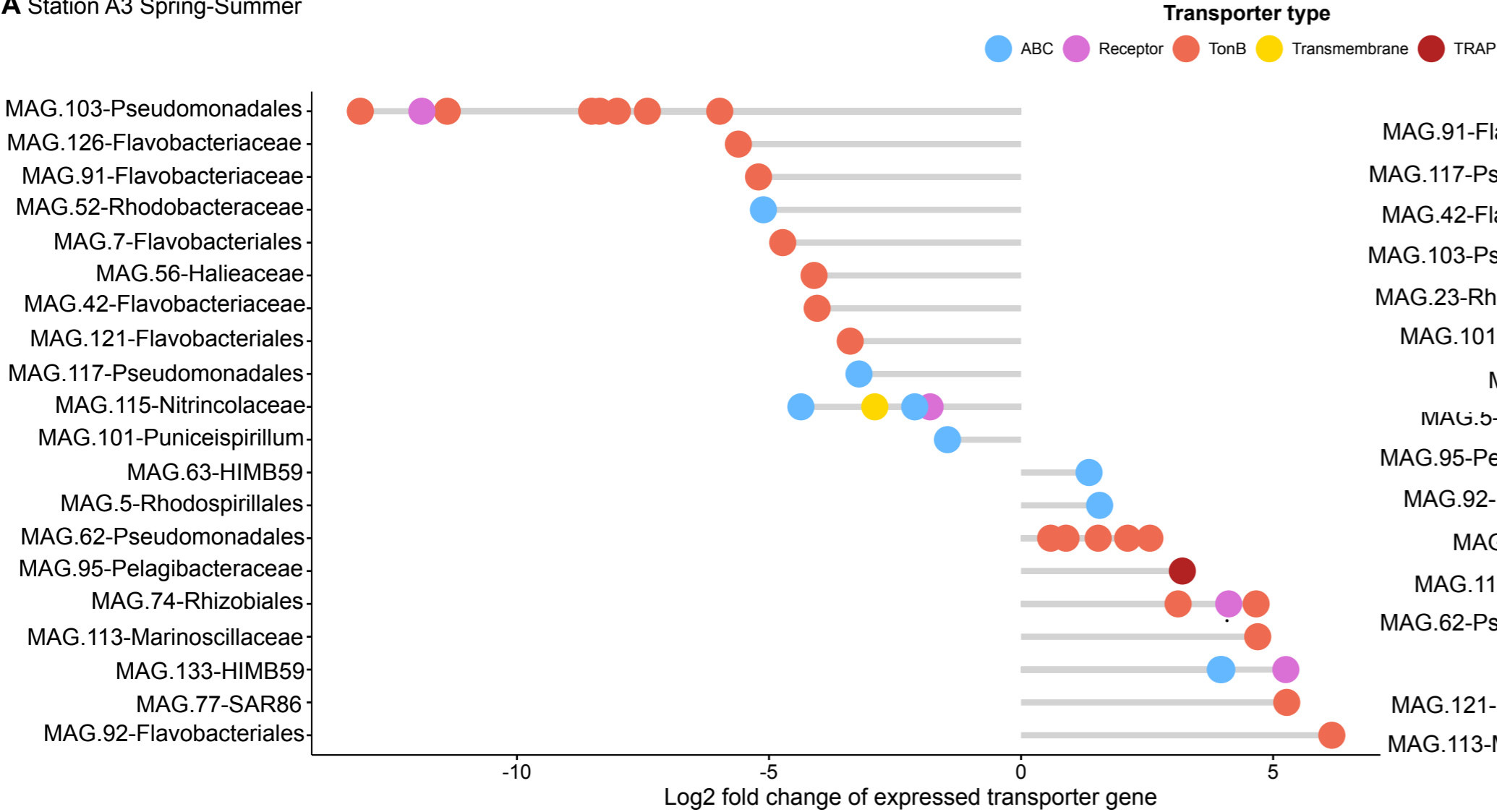

**B** Station KERFIX Spring-Summer

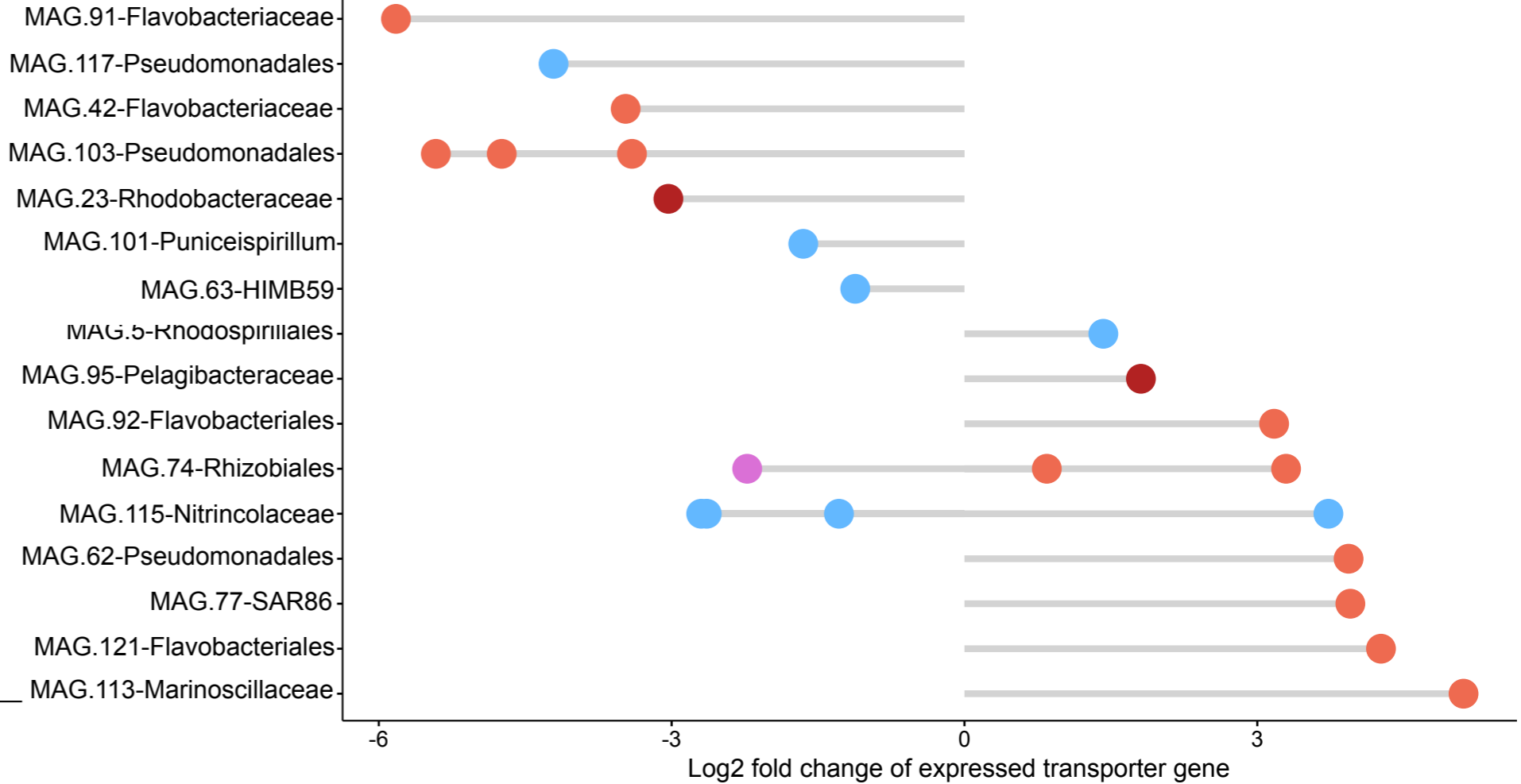

Supplement: Supplementary file 9 — Additional file 8: Supplementary Figure 8. Differentially expressed transporter protein during early spring and late summer for A. Station A3 and B. Station KERFIX. Left side values represent expression in early spring and on the right side, values represent expression in late summer metatranscriptomes by transporter type. MAG Ids show the lowest identifiable phylogenetic level. [file 40168_2023_1600_MOESM8_ESM.pdf]
