# Supplementary material for: Seasonal patterns in microbial carbon and iron transporter expression in the Southern Ocean
Source: Microbiome. 2023 Aug 19;11:187. doi: 10.1186/s40168-023-01600-3 (PMC10439609; doi:10.1186/s40168-023-01600-3)
Supplement: Supplementary file 10 — Additional file 9: Supplementary material. [file 40168_2023_1600_MOESM9_ESM.docx]

**Supplementary material**

**Seasonal differences in microbial carbon and iron utilization revealed by membrane transporters in the Southern Ocean**

Pavla Debeljak^1,2,3^*, Barbara Bayer^2,4^, Ying Sun^1^, Gerhard J. Herndl^2,5,6^

& Ingrid Obernosterer^1^

^1^Sorbonne Université, CNRS, Laboratoire d'Océanographie Microbienne, LOMIC, F-66650 Banyuls/mer, France

^2^University of Vienna, Department of Functional and Evolutionary Ecology, Djerassiplatz 1, 1030 Vienna, Austria

^3^Sup’Biotech, Villejuif, France

^4^University of Vienna, Centre for Microbiology and Environmental Systems Science, Department of Microbiology and Ecosystem Science, Djerassiplatz 1, 1030 Vienna, Austria

^5^ NIOZ, Royal Netherlands Institute for Sea Research, Department of Marine Microbiology and Biogeochemistry, 1790 AB Den Burg, The Netherlands

^6^ Vienna Metabolomics Center, University of Vienna, Djerassiplatz 1, 1030 Vienna, Austria

*** Corresponding author:** Pavla Debeljak, pavla.debeljak@supbiotech.fr

Pavla Debeljak: https://orcid.org/0000-0002-5542-0358

Barbara Bayer: https://orcid.org/0000-0003-3968-5804

Ying Sun: https://orcid.org/0000-0002-4224-341X

Ingrid Obernosterer: https://orcid.org/0000-0002-2530-8111

Gerhard J. Herndl: <https://orcid.org/0000-0002-2223-2852>

**DNA extraction, purification and sequencing**

DNA was extracted from each Sterivex filter unit using the AllPrep DNA/RNA kit (Qiagen, Hiden, Germany) with the following modifications. Filter units were thawed and closed with a sterile pipette tip at the outflow. Lysis buffer was added (40 mM EDTA, 50 mM Tris, 0.75 M sucrose) and three freeze-and-thaw cycles were performed using dry ice in ethanol and a water bath at 65°C. Lysozyme solution (0.2 mg mL^-1^ final concentration) was added and filter units were placed on a rotary mixer at 37°C for 45 min. Proteinase K (0.2 mg mL^-1^ final concentration) and SDS (1% final concentration) were added and filter units were incubated at 55°C with gentle agitation every 10 min for 1 h. To each filter unit, 1550 µl RLT plus buffer was added and inverted to mix. The lysate was recovered by using a sterile 5 ml syringe and loaded in three additions onto the DNA columns by centrifuging at 10,000x g for 30 sec. DNA purification was performed following manufacturer’s guidelines. The concentration of double-stranded DNA was quantified by PicoGreen fluorescence assay (Life Technologies). Triplicate extracts were pooled to equimolar amounts to achieve a final concentration of 1 µg in 30 µL Tris. DNA quality was checked on an Agilent 2100 Bioanalyzer/Agilent Nano DNA chip (Agilent, Santa Clara, CA, USA). Shotgun library preparation was performed by Fasteris SA using the illumina Nano library preparation kit with 550 bp size selection. Each metagenome was sequenced on one full lane of HiSeq 4000 with 150 bp paired-end reads yielding between 285-339 million reads per metagenome.

**RNA extraction, purification and sequencing**

The filtration procedure did not exceed 10 min, and 10 ml of RNA-later was added to the filter in a 15 mL tube prior to storage at -80°C. RNA was extracted using the NucleoSpin® RNA Midi kit (Macherey-Nagel, Düren, Germany). Filters stored in RNA-later were thawed, removed from the RNA-later solution, re-frozen in liquid nitrogen and shattered using a mortar. The obtained ‘powder-like’ filter-pieces were added to the denaturing lysis buffer supplied by the NucleoSpin® RNA Midi kit and vortexed for 2 min. At this step two artificial internal mRNA standards in known copy numbers (MTST5 = 3.70 × 108 molecules; MTST6 = 3.72 × 108 molecules) ~1,000 nt in length were added to each sample individually ([1]; dx.doi.org/10.17504/protocols.io.ffwbjpe). Standards were synthesized using custom templates that were transcribed in vitro to RNA [1]. The extraction with the NucleoSpin® RNA Midi kit includes an on-column DNA digestion step. However, to ensure the absence of any DNA in the sample, a PCR reaction was performed without the re-transcription step. Samples with DNA contamination, as indicated by amplification products were treated with a second DNA digestion step using the Turbo DNA-free kit (Ambion Life Technologies, Carlsbad, CA, USA). This additional DNAse treatment was followed by purification with the RNA Clean & Concentrator™-5 Kit (Zymo Research, OZyme, France). The extracted RNA was quantified with the Agilent 2100 Bioanalyzer/Agilent RNA 6000 Nano Kit (Agilent, Santa Clara, CA, USA). Prior to sequencing, ribosomal RNA was treated enzymatically with the RiboZero rRNA stranded RNA protocol to ensure sequencing of primarily messenger RNA followed by cDNA library construction using Illumina TruSeq Stranded mRNA Library Prep kit (Fasteris SA). All metatranscriptomes (n=12) were sequenced on one lane of HiSeq 4000 with 150 bp paired-end reads yielding between 26-36 million reads per metatranscriptome.

**Metatranscriptomic read quality control**

An initial round of read processing was provided by Fasteris SA using trimmomatic [2], an integrated quality-control tool for high-throughput Illumina NGS data. The standard Illumina adapters and low-quality bases were removed with the following parameters “2:30:10 SLIDINGWINDOW:4:5” and reads for which no insert was found nor ambiguities were removed resulting in million 2×150 bp paired-end reads. The raw Illumina reads were checked with FastQC (https://www.bioinformatics.babraham.ac.uk/projects/fastqc/). An additional quality trimming step was performed with the program Sickle (https://github.com/najoshi/sickle) resulting in between 99.47 - 100% of remaining reads (**Suppl. Table 4**). To focus on protein-coding RNA sequences, we computationally eliminated ribosomal RNA and internal standard-derived reads using the program SortMeRNA v 2.1b [3]. A BLASTN homology search [4] against a custom database, consisting of representative prokaryotic rRNA and tRNA sequences from NCBI RefSeq [5–7] and the template sequences of internal standards, was also implemented using a bit score cut-off of 50 as suggested in previous studies [1, 8]. The files containing protein coding nucleotide sequences derived from SortMeRNA were unmerged and mapped to the reference metagenomic co-assembly using Bowtie2 for read recruitment with default parameters [9]. Overall, 59.8-62.6 % of reads could be mapped to the metagenomic contigs (**Supplementary Table 5**). For read counting the program featureCounts [10] was used with the following parameters for stranded specific reads -F 'GTF' -t 'gene' -Q 1 -s2 -p -C. Count tables were further analyzed using the Likelihood Ratio Test (LRT) as implemented in the DESeq2 software [11] in R [2021, R Core Team]. After correcting for multiple testing, the adjusted P-value < 0.05 was used as threshold to identify transcripts with significant changes in abundance. The raw count data were normalized based on the count data of the internal standards by applying the controlGenes option of the DESeq2 software to statistically test the dynamics of absolute transcript abundance. A further normalization step was performed in order to account for differences in the average feature length of each gene in each sample using the normMatrix option. Transcripts with a total count of less than 50 were eliminated.

**Protein extraction**

Whole protein extractions from filters were performed using a modified protocol [12]. For higher yield of extraction, filters in 15 mL tubes were cooled in liquid nitrogen and smashed with a sterile metal spatula until a powder-like sample of small filter pieces was obtained. Proteins were extracted from filter pieces by adding 10 mL lysis buffer (100 mM Tris-HCl pH 7.5, 150 mM NaCl, 1 mM DTT, 1 % SDS, 10 mM EDTA) to the tubes followed by three freeze-and-thaw cycles and 5 cycles of sonication with a sonication probe (Sonopuls HD 2070, Bandelin) for 30 sec intervals at 20% intensity. Tubes were centrifuged at 20,000x g at 4°C for 10 min and the supernatant containing the cell lysate was collected in a fresh tube. Proteins were co-precipitated with 0.015% deoxycholate and 6% trichloroacetic acid (TCA) on ice for 1h and washed once with ice-cold acetone according to the protocol of Bensadoun and Weinstein [13]. Dried protein pellets were resuspended in 50 µL of 8 M urea containing 4% SDS and protein concentrations were measured with the bicinchoninic acid (BCA) assay using BSA as a standard. Ten-20 µg of protein were subjected to denaturing polyacrylamide gel-electrophoresis (SDS-PAGE), stained with Commassie staining solution and de-stained in 40% (v/v) methanol containing 2% (v/v) acetic acid as described in Valledor and Weckwerth [14]. Gel bands were excised without fractionating the bands, cut into 1 x 1 mm pieces on a sterile glass plate and transferred into protein-low binding tubes (Eppendorf). Gel pieces were de-stained with 200 mM ammonium bicarbonate containing 50% acetonitrile at 37°C for 30 min, and cysteines were reduced and alkylated with 10 mM DTT and 55 mM iodoacetoamide (IAA), respectively, as described by Shevchenko et al. [15] prior to overnight trypsin digestion according to Valledor and Weckwerth [14]. Peptides were extracted and desalted using 96-well plates (Spec 96-Well C18, Agilent) and then resuspended in 2 % acetonitrile/ 0.1 % formic acid to a concentration of 0.2 µg µL-1 prior to injection into a one-dimensional nanoflow LC–MS/MS [14].

**LC-MS/MS analysis and peptide identification**

Five µL of desalinated peptides were eluted using an Easy-spray PepMap RSLC column (ThermoFisher Scientific, C18, 500 mm x 75 µm, pore size 2.0 µm), during a 270-min gradient from 5 to 40% (v/v) acetonitrile and 0.1% (v/v) formic acid with a controlled flow rate of 300 nl min-1. MS analysis was performed on both a Orbitrap Elite and a Q Exactive mass spectrometer (Thermo Fisher Scientific). Specific tune settings for the MS were as follows: Mass resolution for precursor ion analysis in FTMS (Fourier transform mass spectrometry): 60,000; full-scan mode, mass window for precursor ion: 1 m/z; spray voltage: 1.9 kV; temperature of the heated transfer capillary: 275°C, covering the range 350–1,800 m/z, and cyclomethicone used as lock mass (m/z 371.101230). Each full MS scan was followed by 20 dependent MS/MS scans in the Ion Trap using rapid mode with centroid data in which the 20 most abundant peptide molecular ions were dynamically selected. The dynamic exclusion window was set to 30 sec and an exclusion list of 500 entries. Dependent fragmentations were performed in CID (collision-induced dissociation) mode, with a normalized collision energy of 35, iso width of 2.0, activation Q of 0.250, and activation time of 30 msec. Ions with a +1 or unidentified charge state in the full MS were excluded from MS/MS analysis.

Raw peptide spectra were analysed with the software Proteome Discoverer 2.2 (Thermo Fisher Scientific). The mass tolerance was set to 5 ppm for precursor masses and 0.6 Da for the fragment masses. Cysteine carbamidomethylation was set as static-, methionine oxidation and protein N-terminal acetylation as dynamic modifications. A maximum false discovery of 1% was allowed for both peptide and protein levels. Trypsin was specified as the proteolytic enzyme and 2 missed cleavages were allowed and a maximum of 3 equal post-translational modifications (PTMs) per peptide. The obtained peptide spectrum matches (PSMs) were filtered with the Percolator tool [16–19] based on a scoring of maximum delta Cn 0.05, a strict false discovery rate (FDR) of 0.01 with validation based on q-values.

Acquired MS/MS spectra were analyzed using the SEQUEST-HT [20] algorithm implemented in Proteome Discoverer 2.2 software (Thermo Fisher Scientific), and spectra were searched against the translated coding sequences of the corresponding metagenomes (described below in bioinformatic analysis**, Supplementary Figure 2. B&D**). Protein matches were accepted if they were identified by at least one unique peptide and with high confidence. Proteins were quantified using the normalized spectral abundance factor (NSAF) approach [21] as follows:

$${NSAF}_{k}=\left( \frac{PSM}{L} \right)_{k}/\sum_{i=l}^{N} \left( \frac{PSM}{L} \right)_{k}$$

where the total number of spectral counts for the matching peptides from protein k (PSM) was divided by the protein length (L) and then divided by the sum of PSM/L for all N proteins.

**Metagenome analysis**

An initial round of read processing was provided by Fasteris using trimmomatic [2], an integrated quality-control tool for high-throughput Illumina NGS data. The standard Illumina adapters and low-quality bases were removed with the following parameters “2:30:10 SLIDINGWINDOW:4:5”, and reads for which no insert or any or ambiguities were found were excluded resulting in million 2×150 bp paired-end reads. The raw Illumina reads were checked with FastQC (Andrews 2010; http://www.bioinformatics.babraham.ac.uk/projects/fastqc) and decontaminated by running bbduk (BBMap - Bushnell B. - sourceforge.net/projects/bbmap/) with the following parameters k=31 hdist=1 ftm=5 and checked again with FastQC (**Supplementary Table 3**). The program BBNorm was used (parameters: following parameters target=100 min=5 prefilter=t bits=16 ecc=t) to normalize read coverage across samples to a depth of 100 for each library and to facilitate computational effort give the large size of the files (96 – 225 GB) (Bushnell 2014; (**Supplementary Table 3**).

Decontaminated, trimmed and normalized metagenomic sequences were co-assembled using megahit v1.2.7 [22] with the default parameters and the --presets 'meta-large' option resulting in 949,228 contigs of at least 1,000 bp (**Supplementary Table 2&3**). Prodigal, a protein-coding gene prediction software tool for bacterial and archaeal genomes, was used to produce a gene coordinate file (gtf) and a translated nucleotide file with the option -p meta [23].

**Binning for metagenomic assembled genomes (MAGs)**

We applied a combination of the following three binning tools, CONCOCT [24], MaxBin v2.0 [25] and MetaBAT v2.0 [26], to recover genomes from fragmented metagenomic assemblies with a minimum contig size of 2,500 bp. The program, CONCOCT bins the contigs by employing sequence composition and contig coverage across multiple samples [24]. Besides the two features used by CONCOCT, MaxBin also takes advantage of phylogenetic marker genes to perform binning through an Expectation-Maximization (EM) algorithm [25]. MaxBin was performed multiple times with different values for “-markerset” (40 or 107) and “-prob_threshold” (0.5 or 0.8). The MAGs resulting from different parameter combinations were compared based on the completeness and contamination estimations in CheckM [27], by using an R script provided in the MetaBAT workflow ([26] ; https://portal.nersc.gov/dna/RD/Metagenome_RD/MetaBAT/Files/benchmark.R). The parameter set (-markerset 40 –prob_threshold 0.5), which yielded slightly more bins fulfilling given recall (>=0.5) and precision (>=0.9) cutoffs, was selected. All three tools are widely used on current metagenomics studies, but their extents of success vary with characteristics of the underlying datasets [28]. Therefore, we integrated and refined MAGs generated by the three tools by using MetaWRAP, which proposes a scoring function based on the completion and contamination metrics estimated with CheckM [27] . Finally, 133 high-quality MAGs with >= 50% completeness and < 10% redundancy were obtained.

Protein-coding genes of each MAG were retrieved from the before mentioned Prodigal annotations from sequence headers. For phylogenomic inference, 218 single-copy orthologous gene families shared by at least 20 (out of 133) MAGs were identified by OrthoFinder v2.2.3 [29], and aligned with MAFFT v7.313 [30]. Then, the protein sequence alignments of each gene family were concatenated into a super alignment. Maximum likelihood (ML) phylogenetic inference of the 133 MAGs was performed using IQ-Tree v1.6.8 [31] with 1,000 bootstrap replicates, and the amino acid substitution model for each gene family partition was automatically selected by the ModelFinder feature (-m MFP) [32]. Furthermore, taxonomic classification of the 133 MAGs was performed according to the Genome Taxonomy Database using GTDB-Tk v0.3.3 (https://github.com/Ecogenomics/GTDBTk) with the classify_wf workflow [33].

**Protein database construction and annotation of transporter proteins**

Each metagenome was individually assembled using the program megahit and amino acid sequences were predicted using the program prodigal using the same parameters as mentioned above. The command anvi-script-reformat-fasta implemented in Anvi’o [34] was run on each resulting protein fasta file changing header names to include the sample ID for further analysis. Sequences were compared with the program cd-hit-2d [35, 36] using the parameters -c 0.9 -n 5 -d 0 -S2. Additionally, the Global Ocean Sampling (GOS) amino acid sequence database [37] was added. A non-redundant amino acid sequence database was created using cd-hit (-c 1 -n 5 -d 0) resulting in 58,403.522 sequences (**Supplementary Fig. 2**).

In order to identify genes encoding transporter proteins in the individual metagenomic assemblies, predicted amino acid sequences were aligned using the program eggNOG 5.0 [38, 39] and GhostKOALA [40] with default parameters. All KO number defined as “Transporter” in KEGG were retrieved as a database and used for merging with annotated sequences for metatranscriptomes and metaproteomes. Transporter families were verified by manually checking the KO number with assigned classification from the Transporter Classification Database [41–44]. For taxonomic assignments of amino acid sequences to individual MAGs, the sequences corresponding to transporters were aligned against the high quality curated metagenomic bins and those with 95% identity and 90% coverage were kept for further analysis. In order to focus specifically on carbon and Fe utilization we used existing hidden markov models for CAZymes ([45]; http://www.cazy.org/) and Fe transport ([46]; **Supplementary Tables 8&9**).

**Visualization**

All figures were produced using the ggplot2 package in R version 3.6.0 (2019-04-26) [47] and colours were enhanced in the open-source software inkscape. Figure 3 was produced using Anvi’o visualization tool version (5.2.0) in the anvio-interactive manual mode.

**References**

1. Satinsky BM, Gifford SM, Crump BC, Moran MA. Use of Internal Standards for Quantitative Metatranscriptome and Metagenome Analysis. In: Methods in enzymology. 2013. p. 237–50.

2. Bolger AM, Lohse M, Usadel B. Trimmomatic: a flexible trimmer for Illumina sequence data. Bioinformatics. 2014;30:2114–20.

3. Kopylova E, Noé L, Touzet H. SortMeRNA: fast and accurate filtering of ribosomal RNAs in metatranscriptomic data. Bioinformatics. 2012;28:3211–7.

4. Altschul SF, Gish W, Miller W, Myers EW, Lipman DJ. Basic local alignment search tool. Journal of Molecular Biology. 1990;215:403–10.

5. O’Leary NA, Wright MW, Brister JR, Ciufo S, Haddad D, McVeigh R, et al. Reference sequence (RefSeq) database at NCBI: current status, taxonomic expansion, and functional annotation. Nucleic Acids Res. 2016;44:D733-745.

6. Tatusova T, DiCuccio M, Badretdin A, Chetvernin V, Nawrocki EP, Zaslavsky L, et al. NCBI prokaryotic genome annotation pipeline. Nucleic Acids Res. 2016;44:6614–24.

7. Brister JR, Ako-Adjei D, Bao Y, Blinkova O. NCBI viral genomes resource. Nucleic Acids Res. 2015;43 Database issue:D571-577.

8. Gifford SM, Sharma S, Rinta-Kanto JM, Moran MA. Quantitative analysis of a deeply sequenced marine microbial metatranscriptome. The ISME journal. 2011;5:461–72.

9. Langmead B, Salzberg SL. Fast gapped-read alignment with Bowtie 2. Nature Methods. 2012;9:357–9.

10. Liao Y, Smyth GK, Shi W. featureCounts: an efficient general purpose program for assigning sequence reads to genomic features. Bioinformatics. 2014;30:923–30.

11. Love MI, Huber W, Anders S. Moderated estimation of fold change and dispersion for RNA-seq data with DESeq2. Genome Biol. 2014;15.

12. Bayer B, Pelikan C, Bittner MJ, Reinthaler T, Könneke M, Herndl GJ, et al. Proteomic Response of Three Marine Ammonia-Oxidizing Archaea to Hydrogen Peroxide and Their Metabolic Interactions with a Heterotrophic Alphaproteobacterium. mSystems. 2019;4.

13. Bensadoun A, Weinstein D. Assay of proteins in the presence of interfering materials. Analytical Biochemistry. 1976;70:241–50.

14. Valledor L, Weckwerth W. An Improved Detergent-Compatible Gel-Fractionation LC-LTQ-Orbitrap-MS Workflow for Plant and Microbial Proteomics. In: Jorrin-Novo JV, Komatsu S, Weckwerth W, Wienkoop S, editors. Plant Proteomics: Methods and Protocols. Totowa, NJ: Humana Press; 2014. p. 347–58.

15. Shevchenko A, Tomas H, Havlis J, Olsen JV, Mann M. In-gel digestion for mass spectrometric characterization of proteins and proteomes. Nat Protoc. 2006;1:2856–60.

16. Wright JC, Collins MO, Yu L, Käll L, Brosch M, Choudhary JS. Enhanced Peptide Identification by Electron Transfer Dissociation Using an Improved Mascot Percolator. Molecular & Cellular Proteomics. 2012;11:478–91.

17. Brosch M, Yu L, Hubbard T, Choudhary J. Accurate and Sensitive Peptide Identification with Mascot Percolator. J Proteome Res. 2009;8:3176–81.

18. Käll L, Canterbury JD, Weston J, Noble WS, MacCoss MJ. Semi-supervised learning for peptide identification from shotgun proteomics datasets. Nature Methods. 2007;4:923–5.

19. Spivak M, Weston J, Bottou L, Käll L, Noble WS. Improvements to the Percolator Algorithm for Peptide Identification from Shotgun Proteomics Data Sets. J Proteome Res. 2009;8:3737–45.

20. Tabb DL. The SEQUEST Family Tree. J Am Soc Mass Spectrom. 2015;26:1814–9.

21. Zhang Y, Wen Z, Washburn MP, Florens L. Improving Label-Free Quantitative Proteomics Strategies by Distributing Shared Peptides and Stabilizing Variance. Anal Chem. 2015;87:4749–56.

22. Li D, Liu C-M, Luo R, Sadakane K, Lam T-W. MEGAHIT: an ultra-fast single-node solution for large and complex metagenomics assembly via succinct de Bruijn graph. Bioinformatics. 2015;31:1674–6.

23. Hyatt D, Chen G-L, LoCascio PF, Land ML, Larimer FW, Hauser LJ. Prodigal: prokaryotic gene recognition and translation initiation site identification. BMC Bioinformatics. 2010;11:119.

24. Alneberg J, Bjarnason BS, de Bruijn I, Schirmer M, Quick J, Ijaz UZ, et al. Binning metagenomic contigs by coverage and composition. Nat Methods. 2014;11:1144–6.

25. Wu Y-W, Simmons BA, Singer SW. MaxBin 2.0: an automated binning algorithm to recover genomes from multiple metagenomic datasets. Bioinformatics. 2016;32:605–7.

26. Kang D, Li F, Kirton ES, Thomas A, Egan RS, An H, et al. MetaBAT 2: an adaptive binning algorithm for robust and efficient genome reconstruction from metagenome assemblies. PeerJ Inc.; 2019.

27. Parks DH, Imelfort M, Skennerton CT, Hugenholtz P, Tyson GW. CheckM: assessing the quality of microbial genomes recovered from isolates, single cells, and metagenomes. Genome Res. 2015;25:1043–55.

28. Sczyrba A, Hofmann P, Belmann P, Koslicki D, Janssen S, Dröge J, et al. Critical Assessment of Metagenome Interpretation—a benchmark of metagenomics software. Nature Methods. 2017;14:1063–71.

29. Emms DM, Kelly S. OrthoFinder: solving fundamental biases in whole genome comparisons dramatically improves orthogroup inference accuracy. Genome Biol. 2015;16:157.

30. Katoh K, Standley DM. MAFFT Multiple Sequence Alignment Software Version 7: Improvements in Performance and Usability. Mol Biol Evol. 2013;30:772–80.

31. Nguyen L-T, Schmidt HA, von Haeseler A, Minh BQ. IQ-TREE: A Fast and Effective Stochastic Algorithm for Estimating Maximum-Likelihood Phylogenies. Mol Biol Evol. 2015;32:268–74.

32. Kalyaanamoorthy S, Minh BQ, Wong TK, von Haeseler A, Jermiin LS. ModelFinder: Fast Model Selection for Accurate Phylogenetic Estimates. Nat Methods. 2017;14:587–9.

33. Parks DH, Chuvochina M, Waite DW, Rinke C, Skarshewski A, Chaumeil P-A, et al. A standardized bacterial taxonomy based on genome phylogeny substantially revises the tree of life. Nature Biotechnology. 2018;36:996–1004.

34. Eren AM, Esen ÖC, Quince C, Vineis JH, Morrison HG, Sogin ML, et al. Anvi’o: an advanced analysis and visualization platform for ‘omics data. PeerJ. 2015;3:e1319.

35. Fu L, Niu B, Zhu Z, Wu S, Li W. CD-HIT: accelerated for clustering the next-generation sequencing data. Bioinformatics. 2012;28:3150–2.

36. Li W, Godzik A. Cd-hit: a fast program for clustering and comparing large sets of protein or nucleotide sequences. Bioinformatics. 2006;22:1658–9.

37. Yooseph S, Sutton G, Rusch DB, Halpern AL, Williamson SJ, Remington K, et al. The Sorcerer II Global Ocean Sampling Expedition: Expanding the Universe of Protein Families. PLoS Biology. 2007;5:e16.

38. Huerta-Cepas J, Szklarczyk D, Forslund K, Cook H, Heller D, Walter MC, et al. eggNOG 4.5: a hierarchical orthology framework with improved functional annotations for eukaryotic, prokaryotic and viral sequences. Nucleic Acids Res. 2016;44:D286–93.

39. Huerta-Cepas J, Szklarczyk D, Heller D, Hernández-Plaza A, Forslund SK, Cook H, et al. eggNOG 5.0: a hierarchical, functionally and phylogenetically annotated orthology resource based on 5090 organisms and 2502 viruses. Nucleic Acids Res. 2019;47:D309–14.

40. Kanehisa M, Sato Y, Morishima K. BlastKOALA and GhostKOALA: KEGG Tools for Functional Characterization of Genome and Metagenome Sequences. J Mol Biol. 2016;428:726–31.

41. Saier MH, Tran CV, Barabote RD. TCDB: the Transporter Classification Database for membrane transport protein analyses and information. Nucleic Acids Res. 2006;34 Database issue:D181-186.

42. Saier MH, Yen MR, Noto K, Tamang DG, Elkan C. The Transporter Classification Database: recent advances. Nucleic Acids Res. 2009;37 Database issue:D274-278.

43. Saier MH, Reddy VS, Tamang DG, Västermark A. The transporter classification database. Nucleic Acids Res. 2014;42 Database issue:D251-258.

44. Saier MH, Reddy VS, Tsu BV, Ahmed MS, Li C, Moreno-Hagelsieb G. The Transporter Classification Database (TCDB): recent advances. Nucleic Acids Res. 2016;44:D372-379.

45. Lombard V, Golaconda Ramulu H, Drula E, Coutinho PM, Henrissat B. The carbohydrate-active enzymes database (CAZy) in 2013. Nucleic Acids Res. 2014;42:D490–5.

46. Garber AI, Nealson KH, Okamoto A, McAllister SM, Chan CS, Barco RA, et al. FeGenie: A Comprehensive Tool for the Identification of Iron Genes and Iron Gene Neighborhoods in Genome and Metagenome Assemblies. Front Microbiol. 2020;11.

47. Wickham H. ggplot2: Elegant Graphics for Data Analysis. New York: Springer-Verlag; 2009.
